# Metabolic Shades of S-D-Lactoylglutathione

**DOI:** 10.3390/antiox11051005

**Published:** 2022-05-20

**Authors:** Miklós Péter Kalapos, Cinzia Antognelli, Lidia de Bari

**Affiliations:** 1 Theoretical Biology Research Group, Dámvad utca 18, H-1029 Budapest, Hungary; 2 Department of Medicine & Surgery, Bioscience and Medical Embryology Division, University of Perugia, L. Severi Square, 06129 Perugia, Italy; cinzia.antognelli@unipg.it; 3 Institute of Biomembranes, Bioenergetics and Molecular Biotechnologies-CNR, Via G. Amendola 122/O, 70126 Bari, Italy; l.debari@ibiom.cnr.it

**Keywords:** S-D-lactoylglutathione, methylglyoxal, glyoxalases, N-lact(o)ylation, S-glutathionylation, cytoskeleton

## Abstract

S-D-lactoylglutathione (SDL) is an intermediate of the glutathione-dependent metabolism of methylglyoxal (MGO) by glyoxalases. MGO is an electrophilic compound that is inevitably produced in conjunction with glucose breakdown and is essentially metabolized via the glyoxalase route. In the last decades, MGO metabolism and its cytotoxic effects have been under active investigation, while almost nothing is known about SDL. This article seeks to fill the gap by presenting an overview of the chemistry, biochemistry, physiological role and clinical importance of SDL. The effects of intracellular SDL are investigated in three main directions: as a substrate for post-translational protein modifications, as a reservoir for mitochondrial reduced glutathione and as an energy currency. In essence, all three approaches point to one direction, namely, a metabolism-related regulatory role, enhancing the cellular defense against insults. It is also suggested that an increased plasma concentration of SDL or its metabolites may possibly serve as marker molecules in hemolytic states, particularly when the cause of hemolysis is a disturbance of the pay-off phase of the glycolytic chain. Finally, SDL could also represent a useful marker in such metabolic disorders as diabetes mellitus or ketotic states, in which its formation is expected to be enhanced. Despite the lack of clear-cut evidence underlying the clinical and experimental findings, the investigation of SDL metabolism is a promising field of research.

## 1. Introduction

**Figure 1:**
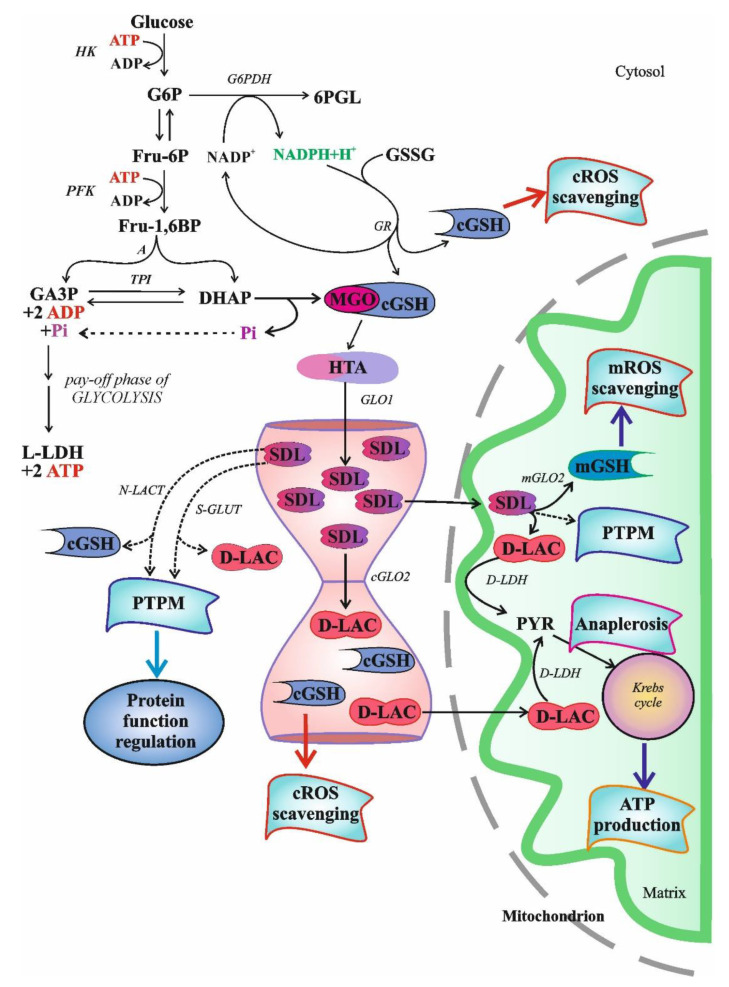
The interactions of the glyoxalase system with cell energy metabolism, the antioxidant system and protein function regulation. MGO formation and elimination by the glyoxalase pathway is graphically described as an integral part of cell metabolism. The routes by which SDL could contribute to cell energy production, ROS scavenging and protein function regulation are highlighted. The hourglass stresses the fact that GLO2 activity is the rate-limiting step of the MGO pathway: this could be relevant, under certain conditions, such as for an SDL level increase in the cytosol, which could favor its utilization in additional paths, such as PTPM and mitochondrial GSH pool replenishing (see the text for details). Abbreviations: GR, glutathione reductase; cGSH, cytosolic reduced glutathione; mGSH, mitochondrial reduced glutathione; GSSG, oxidized glutathione; MGO, methylglyoxal; HTA, hemithioacetal; SDL, S-D-lactoylglutathione; GLO1, glyoxalase I; cGLO2, cytosolic glyoxalase II; mGLO2, mitochondrial glyoxalase II; D-LAC, D-lactate; D-LDH, D-lactate dehydrogenase; ROS, reactive oxygen species; mROS, mitochondrial ROS; cROS, cytosolic ROS; PTPM, post-translational protein modification; N-LACT, N-lactoylation; S-GLUT, S-glutathionylation; glycolysis intermediates, cofactors and enzymes related to glycolysis are not listed.

S-D-lactoylglutathione (SDL) is an intermediate in the glyoxalase pathway (Figure 1). The glyoxalase route comprises two enzymes, designated glyoxalase I (GLO1) and glyoxalase II (GLO2), uses reduced glutathione (GSH) as cofactor and catalyzes two consecutive steps in the GSH-dependent metabolism of methylglyoxal (MGO) [1,2]. Up to the present day, the glyoxalase route is the only known intracellular source of SDL.

While much has been learned about MGO metabolism and its actions are under active investigation, particularly focusing on its role in post-translational protein modification (PTPM), little is known about SDL. A comprehensive work on this topic has not been published yet. There are several reasons for this; the most important is likely the fact that the role(s) of SDL in biochemical machinery is (are) far from being understood, whereas the examination of its participation in PTPM is an expanding field of interest [3].

The current overview of the data enumerates the key discoveries made on SDL over the last decades. It concentrates on the chemistry, metabolism and toxicity of SDL, along with a brief review of its possible role in evolution and diseases. In order to avoid unnecessary repetitions of the literature, when it is appropriate, the reader is directed to pertinent articles to keep the focus upon the topic intended to be discussed.

## 2. Chemistry, Preparation and Biochemistry of S-D-Lactoylglutathione and Its Measurement in Biological Samples

### 2.1. Chemistry

SDL possesses a thioester bond. During the hydrolysis of this bond, as many as 11.24 kcal/mol (49.23 kJ/mol) of energy is liberated, and the rate constant of the reaction is k = 14.9 ± 1.1 M^−1^s^−1^ at pH 7.4 [4,5]. It is quite stable in an acidic milieu but decomposes into D-lactic acid and GSH on the basic side of neutrality [6,7]. Therefore, when stored at −20 °C at pH 3 to 6, it is stable for several months, while its half-lives at pH 7.4 are 3.7 and 1.1 days at 25 °C and 37 °C, respectively [5,8]. Its hydrolysis follows first order kinetics with respect to hydroxide ion concentration in the pH range of 7 to 9 and also with respect to SDL concentration but seems to be independent of the phosphate ion content of the incubation medium [5]. It is also stable against iodine, a feature that characteristically distinguishes it from the hemithioacetal (HTA) formed from MGO and GSH, as the latter is decomposed by iodine [6].

SDL gives a high light absorption in the low ultraviolet region, with a maximum at a wavelength of 235 nm [7]. For its determination, the measurement at a wavelength of 240 nm is used, and the absorption coefficient at this wavelength is not affected by small changes in pH but falls off when the milieu becomes basic [7]. Like other thioesters formed in the presence of GLO1 from ketoaldehydes and GSH, SDL also gives a characteristic color reaction with hydroxylamine and ferric chloride, a reaction similar to that given by acyl phosphates [8,9].

### 2.2. Preparation

The first and very sophisticated method for SDL preparation, named biological compound at that time, was described by Yamazoye [6]. Racker used GLO1 to prepare SDL in combination with several washing, centrifugation and drying steps [7]. To increase the yield, either GLO1 bound to Sepharose 4B or genetically engineered *Escherichia (E.) coli* and glycerol-grown *Saccharomyces (S.) cerevisiae* were used [10,11]. The yield of the enzymatic method combined with the application of a Dowex-1 column was about 90% [8]. Later, a large-scale enzymatic synthesis and purification of S-2-hydroxyacylglutathione derivatives, including SDL, was published [12].

A two-step synthesis of S-D-[2-^14^C] lactoylglutathione from [2-^14^C] acetone was also reported [12]. The labeled acetone was first oxidized by selenium dioxide to MGO, followed by the enzymatic conversion of the resulting α-oxoaldehyde to SDL. The formed SDL was purified by anion exchange chromatography with a final yield of as much as 82%.

Nowadays, SDL is manufactured and is commercially available.

### 2.3. Biochemistry

#### 2.3.1. S-D-Lactoylglutathione Formation and Degradation

Whatever way MGO is generated, it is mainly detoxified by the ubiquitous glyoxalase system, which is capable of converting not only MGO but also other α-oxoaldehydes into their α-hydroxy-carboxylic acid counterparts [1,2,13,14,15]. The glyoxalase system is present in the cytosol and comprises two enzymes, GLO1 and GLO2 (Figure 1.).

##### GLO1 (S-Lactoylglutathione Methlylglyoxal Lyase, EC 4.4.1.5)

GLO1 acts on the HTA formed from MGO and GSH and catalyzes a one-substrate isomerization of this hemimercaptal into SDL [1,2,16,17,18,19]. Until today, it has been widely held that HTA is exclusively formed spontaneously from MGO and GSH, but according to a recent report, a glutathione S-transferase isozyme in *Synechocystis sp*. strain PCC 6803 catalyzes this conjugation [20]. As to the mechanism, isomerization via an enediol intermediate and a shielded proton transfer are crucial [1,17,21]. The latter step, the proton abstraction by a glutamate residue from the C1 carbon, is believed to be the rate-limiting step in the reaction [22]. GSH is a competitive inhibitor of the enzyme [21].

Despite the generally accepted mechanism, a study based on the analysis of kinetic data gained on yeast GLO1 showed that the results fit better when it was assumed that a GSH-GLO1 complex formed first and was followed by MGO binding [23]. Although this observation needs corroboration, it may reopen the debate on the nature of the real GLO1 substrate.

The kinetic parameters, the k_cat_ and K_M_ values, for the forward reaction in the case of the human red blood cell enzyme are 3.5 × 10^4^ min^−1^ and 57 μM, respectively [1,24]. The corrected values for the reverse reaction catalyzed by GLO1 from human erythrocytes with SDL as substrate are k_cat_ = 108 min^−^^1^ and K_M_ = 1.9 mM [1,24]. Nevertheless, the reversibility of the GLO1 reaction is probably without any real physiological significance, as it may only occur at low GSH concentrations and SDL is hydrolyzed by the action of GLO2 [24].

Since GLO1 ablation prevents SDL production, its formation is solely dedicated to this enzyme and correlates with increased glucose uptake [25,26]. Nevertheless, non-enzymatic formation of SDL, at least in vitro, is also possible. The HTA is spontaneously converted to SDL in a reaction that is enhanced by hydrogen phosphate anions and imidazole [27].

##### GLO2 (S-2-Hydroxyacylglutathione Hydrolase, EC 3.1.2.6)

GLO2 splits SDL into D-lactate and GSH, thereby regenerating the GSH consumed in the GLO1-catalyzed reaction (Figure 1) [1,2,17,19]. The nucleophilic attack of SDL by a histidine residue of the enzyme results in D-lactate formation and the recovery of GSH [1,17,21,28]. A feed-forward inhibition for GLO2 is seen since both GSH and HTA are inhibitory to the enzymatic activity [16,28,29].

The kinetic parameters, the k_cat_ and K_M_ values, for GLO2 from rat erythrocytes are 1.7 × 10^4^ min^−1^ and 180 μM, respectively [1,30]. The k_cat_ value for the rat erythrocyte enzyme seems to be pH-independent in a relatively wide range (5.5–9.5) and solvent-insensitive, while the human liver enzyme is more pH-sensitive (6.8–7.5) [1]. A reversal of the reaction does not seem to be possible due to the high free-energy barrier [4].

Recently, the description of both the association of GLO2 with the proapoptotic BAX protein in non-small cell lung cancer and the function of this complex in the regulation of apoptosis has assigned a new non-enzymatic role to GLO2 [31].

Unlike GLO1, GLO2 activity is present both in the cytosol (cGLO2) and in the mitochondria (mGLO2) of spinach leaves, *Arabidopsis thaliana, Brassica juncea, S. cerevisiae* and rat liver and brain [32,33,34,35,36,37,38,39,40,41]. cGLO2 accounts for about 85–90% of total GLO2, with the remainder being mGLO2 [39]. Interestingly, *Plasmodium falciparum* also harbors two GLO2 isozymes, and one of them is located in the apicoplast [42]. Using a polyclonal antibody against GLO2, intense staining has recently been documented in the nuclei of human prostate cancerous cells but not of their normal counterparts [43].

In addition to GLO2, there are other enzymes capable of handling SDL.

##### sFGH (S-Formylglutathione Hydrolase, EC 3.1.2.12)

The enzyme of both prokaryotic and eukaryotic origin is known, and its function is to split S-formylglutathione to GSH and formate [44,45,46]. There is evidence according to which sFGH is identical to esterase-D in humans [47,48]. The kinetic parameters, the k_cat_ and K_M_ values, for the sFGH prepared from *E. coli* are 0.05–1.02 s^−1^ and 600 μM with SDL as substrate, respectively [45]. 

##### γGT (γ-Glutamyl-Transpeptidase, EC 2.3.2.2.)

The function of γGT is to manage the transfer of the γ-glutamyl moiety of S-substituted GSH derivatives to amino acid or peptide acceptors [49,50,51]. SDL is also a substrate for the enzyme [49]. The enzyme is predominantly membrane-bound and localized on the external surfaces of cells [50]. However, it is also found in the cytosol of certain cells and in human blood plasma, and it is expressed to varying degrees in almost all cells [50,52,53]. Since it accepts SDL as substrate, it could interfere with GLO2 assays [54].

##### Paroxysmal Non-Kinesigenic Dyskinesia (PNKD) Proteins

The PNKD protein encoded by the myofibrillogenesis regulator 1 (MR-1) gene has a conserved enzymatic domain, the C-terminal β-lactamase. Thus, it belongs to the human metallo-β-lactamase fold protein (HMBLf) superfamily [55,56,57,58]. The group of these enzymes, known as the GLO2 subfamily, harbors GLO2, PNKD and hydroxyacylglutathione hydrolyse-like protein (HAGHL) [57,58]. At least three confirmed alternate splice isoforms, named PNKDL (MR-1L), PNKDM (MR-1M) and PNKDS (MR-1S), with this conserved domain and possible biological functions have been identified, while a fourth one that lacks the mentioned domain has not been identified without a doubt [55,56,57,58,59]. In contrast to the ubiquitously expressed PNKDM and PMKDS that are present in both the cytoplasm and nucleus, PNKDL is specifically expressed in the brain and is localized to the cell membrane [55,59,60]. It is homologous to GLO2 and has the ability to hydrolyze SDL at a low rate [55,56]. When phosphorylated, it activates the MAPK signaling pathway, thus functioning as a tumor promoter [60]. Transgene experiments, however, indicated that it did not rescue the lost GLO2 activity. Thus, it is unlikely to be relevant in SDL metabolism in vivo [56]. Therefore, it has been suggested that another, yet unknown, α-hydroxythioester may be its substrate [56]. Even though the function of PNKD is unclear, it appears that PNKD can play a role in fiber formation (cell proliferation and neurotransmitter release) and in the regulation of cellular redox status [56,57,58,60].

Finally, SDL is not a substrate for the non-specific GSH transporter [29].

Beside enzymatic degradation, the non-enzymatic hydrolysis of SDL is also possible, and the magnitude of the rate constant for spontaneous hydrolysis is 10^6^ times lower than the rate constant for GLO2 [4]. 

#### 2.3.2. Regulation of Glyoxalases, with Glyoxalase II in the Focus

The regulation of glyoxalases appears at the levels of transcription, translation and post-translation, but more data are available for GLO1 than GLO2 [19,61,62,63,64]. 

In HT22 nerve cells, NRF2 (nuclear factor-erythroid 2 p45 subunit-related factor 2, a transcription factor that regulates detoxifying and antioxidant defense gene expression) was induced throughout the 24-h experiment by MGO in a concentration-dependent manner up to 0.3 mM, while 0.75 mM MGO had an inhibitory effect at the beginning and an inductive effect developed later, and the induction by lower dicarbonyl levels slowly faded away [65]. GLO1 expression peaked at 0.5 h when 0.3 mM MGO was added and then decreased back to the control level in such a way that its activity remained unchanged [65]. The induction of GLO2 peaked for a longer period of time, then decreased first and afterwards increased again but only in the case of 0.3 mM MGO [65]. MGO was able to initiate the transcriptional activation of both GLO1 and GLO2 in a dose-dependent manner, and this activation was linked to NRF2 activation [65]. On the contrary, NRF2 silencing was unable to repress GLO2 upregulation in androgen-responsive prostate cancerous cells, while androgen receptor silencing abolished GLO2 upregulation but not that of NRF2 [43]. Collectively, these experiments revealed that the effects may even be the opposite, depending on the concentration of dicarbonyl as well as cell type, and that the time windows for GLO1 and GLO2 differ.

The gene encoding GLO2, designated HAGH or GLX2, harbors a response element in intron 1 that can be activated and bound by the p53 family members p63 and p73, resulting in upregulation at the transcriptional level and protein overexpression of cGLO2 [66]. In addition, GLO2 was decreased in the temporal cortex of p53^−/−^ mice [67]. A negative correlation was, however, described between p53 and GLO2 in prostate tumor cells [43], stressing that the members of this transcription factor family have distinct functions in GLO2 regulation that differ in normal and malignant cells. 

The addition of tumor-promoting phorbol-diester (phorbol 12-myristate 13-acetate, TPA, an activator of protein kinase C) to human leukocytes or to *S. cerevisiae* increased GLO1 and decreased GLO2 activities, resulting in a possibility for the intracellular accumulation of SDL [68,69,70,71]. Activated zymosan (protein–carbohydrate complexes prepared from yeast cell wall to induce sterile inflammation) performed similar effects upon glyoxalase activities in human neutrophils, concomitant to an increase in SDL and a decrease in intracellular GSH levels [69,72]. In the case of *S. cerevisiae,* the mating factor by binding to a-factor receptors led to similar changes in GLO1 and GLO2 activities, and the phosphorylation of GLO1 was detected [73,74,75]. The phosphorylation of GLO1 in other systems was also documented, and a threonine residue (Thr-106) was the target motif [76,77,78]. Interestingly, GLO2 phosphorylation was not investigated in either of the cases. Very recently, a protein kinase A-dependent hormone-regulated phosphorylation of both GLO1 and GLO2 was identified in porcine ampulla and primary cell cultures, but the enzyme activities were not revealed [79].

The regulation of glyoxalases by NO was first raised in 1999 [80]. The S-nitrosylation of GLO2 has not been examined in mammals. In plants, the addition of sodium nitroprusside, an NO donor, either increased or decreased GLO2 activity, depending on which plant was used in the experiments [81,82].

S-glutathionylation was documented for cGLO2 in malaria parasites [83]. On the contrary, GLO2 from trypanosomatids proved to be insensitive to S-glutathionylation [84].

#### 2.3.3. Thiols Replacing Glutathione in Glyoxalase Function

In trypanosomes (*Trypanosoma cruzi* and *Leishmania donovani*), the GSH/GSH-reductase system is replaced by the trypanothione (T[SH]_2_)/trypanothione-reductase system [85]. In these parasites, in contrast to all other organisms investigated, T[SH]_2_ is the preferred substrate for the glyoxalase system [86]. While GLO1 also catalyzes the isomerization of the GSH-derived HTA, GLO2 exclusively uses T[SH]_2_-derived thioester [86]. In addition, the GLO1 enzyme is missing from both *Cestodes* and *Digeneas* as well as *Trypanosma brucei* but it is present in *Nematodes* and also in *Leischmania donovani* and *Trypanosoma cruzi* [87,88].

In Gram-positive bacteria, such as *Bacillus (B.) subtilis*, bacillithiol (BSH) is a structurally distinct low-molecular-weight (LMW) thiol that serves metabolic functions similar to GSH [89]. However, glyoxalases using BSH have not yet been thoroughly characterized. Nevertheless, it has been reported for *B. subtilis* that S-lactoyl-BSH played a role in the activation of the K^+^ efflux system, a function well-documented for SDL in *E. coli* [90,91]. Another LMW thiol is mycothiol, which probably also has a function as a cofactor for glyoxalases in *Actinomycetes*, but even less is known about it than about BSH [92,93]. All in all, LMW thiols play a role in the protection of cells against dicarbonyls.

Finally, the GSH-independent glyoxalase III (GLO3), belonging to the DJ-1 proteins, is essentially a deglycase, whereas its ability to convert MGO to lactate, an activity that is 1000-fold lower than that of the glyoxalase route, is a simple reflection of its main activity [94,95,96]. Recently, it has emerged that MGO bypassing the deglycase path would have directly been converted to lactate by this enzyme [97]. Despite uncertainties related to the mechanism, GLO3 does not participate in SDL metabolism but plays a role in the cellular defense against dicarbonyl glycation, thus indirectly influencing SDL formation [96].

### 2.4. The Measurement of S-D-Lactoylglutathione in Biological Samples

Historically, Racker detected SDL by measuring its absorption at a wavelength of 240 nm [7,9]. In the last two decades, two kinds of methods have essentially been used to detect SDL in biological samples. Both involve the deproteinization of samples. First is a two-step spectrophotometric assay of the chromophore conjugate of GSH and 1-chloro-2, 4-dinitrobenzene, while the second involves the application of an HPLC instrument. The detection of absorption at a wavelength of 240 nm was also used to determine the MGO and GSH concentrations of the samples [98].

In the first method, an aliquot of an N-ethylmaleimide-treated (to remove native GSH) and deproteinized sample is first combined with purified GLO2 and then the released GSH is measured. To detect GSH, 1-chloro-2, 4-dinitrobenzene is added to the sample, and the reaction is started by the addition of a suitable amount of glutathione S-transferase (EC 2.5.1.18). The formed chromophore S-2, 4-dinitrophenylglutathione is monitored at a wavelength of 340 nm [99,100]. The concentration of SDL measured in this way has been 12.4 ± 4.8 μM in human blood [100]. This procedure has been mainly used to determine SDL in in vitro experiments with tumor cells, leukocytes and isolated murine hepatocytes [25,69,72,101,102,103,104].

The common feature of the second family of methods is the application of HPLC. After the deproteinization of samples, either an anion-exchange solid-phase extraction was used prior to reverse-phase HPLC [105] or SDL was simply separated and quantified using an isocratic HPLC procedure [106]. Recently, a column-switching HPLC method with a precolumn fluorescence derivatization with 4-fluoro-7-nitro-2,1,3-benzoxadiazole, a fluorogenic reagent, has been worked out in which the deproteinization of the samples is also essentially involved [107]. In healthy humans, the SDL concentration of blood measured by HPLC has been found to be as high as 16.5 ± 4.4 μM [101]. HPLC-based methods have been used to detect SDL concentrations in normal human as well as rat blood and in promyelocytic leukemia cells [106,107,108,109,110,111].

It should, however, be noted that the blood SDL data originating from GLO2-based assays are disputed and are believed to be overestimations of the amounts, probably reflecting at least two factors. The first factor is the acid used to denature the samples, as it is critical to the acid-catalyzed ester formation occurring between GSH and L-lactate, which is abundant in the samples and between GSH and D-lactate [105,112]. Since GLO2 accepts both L- and D-stereoisomers of the substrate, the discrepancy is obvious [13]. The second factor might be the release of SDL from non-covalent complexes formed with intracellular proteins [113]. This is more of a theoretical opportunity that has not yet been investigated.

An ^1^H NMR assay for the examination of SDL is also available in the literature [29]. 

## 3. Physiological Role of S-D-Lactoylglutathione

Although GLO2 is very active, its catalytic efficiency is an order of magnitude smaller than that of GLO1 (see k_cat_ values above) [1,24,30,113]. The measured V_max_ values for GLO1 are usually higher than those of GLO2 in in vitro experiments [16]. These raise the opportunity for an increase in intracellular SDL concentration.

Under physiological conditions, when both HTA and SDL concentrations are small, in other words they are below the K_M_ values, the hydration of MGO is unimportant, the apparent rate constants for GLO1 and GLO2 are equal, the rate-limiting step is the formation of HTA itself and GLO1 activity is proportional to the cytosolic GSH level, as there is a rapid equilibrium between MGO plus GSH and HTA [4,114]. Hence, SDL accumulation in the cells is not very feasible under these circumstances, particularly in light of the fact that at low GSH GLO1 is also able to catalyze the reverse reaction [24]. Thus, the overall velocity depends on GSH availability, and a declining cellular GSH concentration goes together with an impaired GLO1 activity [114]. 

Nevertheless, another point needs to be added. In the presence of GSH, aldose reductase (EC 1.1.1.21) (AR) functions as a ketone reductase. Thus, the efficacy of MGO reduction by the enzyme to lactaldehyde increases [115]. However, when the intracellular GSH concentration is below normal, the metabolic importance of AR in the disposal of 1,2-dicarbonyl exceeds that of the glyoxalase route and, instead of catalyzing lactaldehyde formation, it catalyzes acetol formation [115]. If this is the case, then acetol can be converted back to MGO either by an oxidation governed by CYP2E1 isozymes or by undergoing a disproportionation in the presence of copper ions without the participation of any enzyme [115].

Indeed, the accumulation of SDL is only possible when substrate concentrations exceed K_M_ values since in this case the velocity of GLO2 becomes the upper limit of conversion [4]. Hence, an MGO load, as its uptake is very fast, may easily lead to an accumulation of SDL and to a decrease in GSH [104,116].

### 3.1. The Role of Intracellular S-D-Lactoylglutathione

#### 3.1.1. Post-Translational Protein Modifications

PTPMs represent a family of mechanisms that use a wide variety of substrates and by which the physiological role of a protein can be modulated by covalent modification at specific sites in the majority of cases in a reversible manner [117].

**Figure 2:**
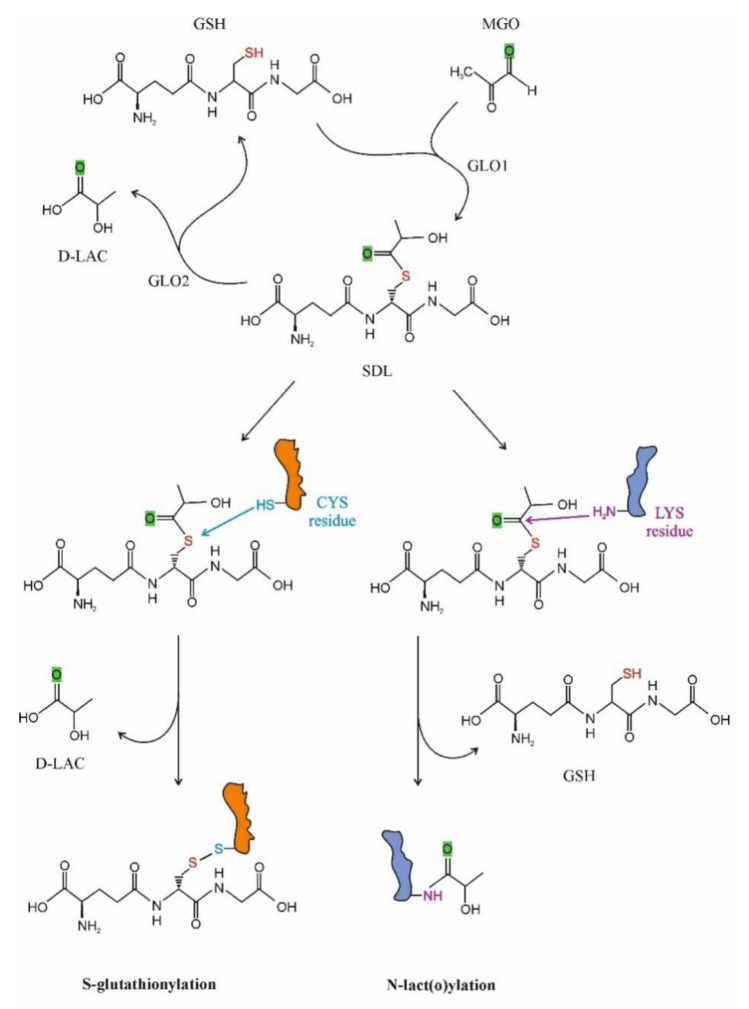
S-glutathionylation and N-lact(o)ylation by S-D-lactoylglutathione. Abbreviations: MGO, methylglyoxal; GSH, reduced glutathione; SDL, S-D-lactoylglutathione; GLO1, glyoxalase I; GLO2, glyoxalase II; D-LAC, D-lactate; CYS, cysteine; LYS, lysine.

Recently, interest is increasing in SDL as a protein-modifying molecule. Two possible ways for PTPM by SDL have been recognized: S-glutathionylation and N-lact(o)ylation (Figure 2). In the first case, the cysteine residues of the given proteins are targeted, while in the latter case lysine motifs are lact(o)ylated.

Regulation through S-glutathionylation has been ascribed to a large number of proteins that fall into the following clusters: cytoskeletal, glycolysis/energy metabolism, kinase and signaling pathways, calcium homeostasis, antioxidant enzymes and protein folding [92,118,119,120,121,122,123,124]. Several enzymes participate to the process that can be either reversible or irreversible, the latter being implicated in diseases and aging [3,125].

For the first time in 2004, the cGLO2 enzyme was documented as a potential candidate to promote S-glutathionylation in vitro [126]. Subsequent studies revealed that GLO2 managed a rapid and specific protein-SSG formation, including actin [126,127]. Nevertheless, it is noteworthy that the solely used SDL was also able to S-glutathionylate proteins [127,128]. As it turned out, the Cys^374^ residue of the actin was S-glutathionylated, and the presence of GLO2 and SDL led to higher S-glutathionylation of the protein than SDL or GSH alone [128]. The effect of SDL on actin S-glutathionylation was dose-dependent [128]. A striking feature of the events was that GLO2 activity was lowered in the presence of actin, and GSH stabilized the GLO2 and actin complex [126,128,129]. Microfilaments, also named actin filaments, are, with rare exceptions, found in the cytoplasm of all eukaryotic cells, where they represent an essential component of the cell cytoskeleton [130]. They are constructed by the combination of G (globular)-actin monomers forming polymers that are then assembled into two intertwined chains named F (filamentous)-actin [131]. The EGF (epidermal growth factor)-provoked S-glutathionylation of actin slowed down the polymerization of actin in A431 epidermal cells, as the polymerization of deglutathionylated protein was six-fold higher than that of glutathionylated protein [132]. The residue of interest was, again, Cys^374^ [132]. The increase in actin S-glutathionylation at Cys^374^ impaired the chemotaxis, adhesion and phagocytosis of murine and human neutrophiles as a result of S-glutathionylated G-actin formation, and ROS depletion led to a higher amount of F-actin that could polymerize [133].

As seen for glyoxalases, other LMW thiols, BSH and mycothiol also participate in S-thiolation in bacteria, and the pathways show a high degree of similarity [88]. An interesting aspect of redox regulation, however, is the presence of coenzyme-A in both bacterial and eukaryotic systems, raising the question of why it is so strictly conserved in the course of evolution.

It currently appears that the role of GLO2 in PTPM is not restricted to S-glutathionylation because it also participates in the N-acetylation of lysine residues in such a way that it limits the intermediate protein S-acetylation [134]. 

N-lact(o)ylation is a recently described type of acylation, a new member of a long-known family of PTPMs [135]. The non-enzymatic acyl transfer of the lactate moiety from SDL to the lysine residues of the cysteine-free histone H4 as well as the N-lact(o)ylation enriched by the SDL of glycolytic enzymes have recently been demonstrated [136]. The role of SDL in the process was further substantiated by the observation that this transfer increased in GLO2 knockout mice, while it was not detected in GLO1^−^ counterparts. The addition of MGO enhanced PTPM [136]. Thus, we could speculate that due to the bottleneck represented by the GLO2 reaction, metabolic conditions leading to increased SDL formation and a consequent SDL level increase in the cytosol could favor its utilization in alternative ways, including protein function regulation via PTPMs, resulting in cell adaptation to the ongoing condition (Figure 1).

In the case of histone N-lact(o)ylation, the nature of the lact(o)yl-group donor is, however, a subject of debate. Although lact(o)yl-coA has been designated as an acyl donor, others dispute this, and SDL is proposed as an acyl donor [137,138,139]. On the basis of experiments with inhibitors applied in the original reports, the nature of an acyl donor cannot be ascertainable, as the inhibition of the pay-off phase of glycolysis equally elevates the levels of both acyl-thioesters [137,138,139].

#### 3.1.2. Cytoskeleton Assembly

The cytoskeleton is a dynamic network of interlinked protein filaments present in the cytoplasm of cells that is composed of three main components: microfilaments, intermediate filaments and microtubules. It functions as the mechanic machinery for the cells and is capable of growing or disassembling, depending on the actual cellular requirements. The cytoskeleton participates in secretion, cytokinesis and movement, intracellular transport and changes in cell shape.

Microtubules are built up from similar protein subunits, α- and β-tubulin, and these αβ dimers polymerize, a step inhibited by colchicine [140]. These dimers bind two GTPs, one tightly, while the other is less tightly bound, and there are several microtubule-associated proteins (MAPs) regulating their stability [130,140,141].

The investigation of the association of MGO and its intermediate SDL with microtubules is not the field of current interest. Only a few communications, exclusively on MGO, are available in this regard. The arginyl residues of tubulin play an essential role in tubulin polymerization through an interaction with the negatively charged phosphate moiety of GTP [142]. In addition, 2, 3-butanedione, an arginyl-residue-modifying agent, prevents microtubule formation from tubulin [142]. MGO also modifies the arginyl residues of proteins [143]. Hence, it is perhaps appropriate to suggest that microtubule formation would also be affected in some way by MGO. MGO decreased the sulfhydryl content as well as the polymerization of microtubular proteins, but these effects seemed to be separated [144]. In addition, proteins from tumors were more resistant to the oxoaldehyde than those originating from normal tissues, and the polymerized forms were comparatively less sensitive than the soluble form [144]. On the contrary, MGO facilitated actin polymerization at low concentrations (0.1–10 μM), both in purified systems from rabbit skeletal muscle and in cell extracts of Ehrlich ascites tumor cells [145]. MGO, similar to but at different rate than other aldehydes with the exception of 2, 3-butanedione, blocked the binding of colchicine to tubulin [142,144,146].

For SDL, even less is known. Only a suggestion of the participation in and the release from the pool of non-covalent complexes formed with intracellular proteins is available [112]. Nevertheless, the actions of externally added SDL were examined (see below).

For glyoxalases, notably for GLO2, some data are accessible.

In 1975, it was noted that MGO and SDL (both at a concentration higher than 1 mM) as well as methylglutathione and GLO2 inhibited microtubule assembly in rat brain supernatants, while GLO1 did not have any effect [147]. In the case of purified tubulin, the experiments with SDL led to contradictory results [147].

In the case of 1 mg/mL microtubular protein (80% tubulin and 20% MAP) in the presence of 20 μM GTP, SDL increased microtubule assembly in a concentration-dependent manner in a cell-free system [101]. When purified porcine brain microtubular proteins were used, SDL added in a concentration between 1 μM and 2 mM potentiated GTP-promoted microtubule assembly [148]. This ability of SDL remained, even after the third aggregation/disaggregation cycle, but this effect disappeared in the presence of GLO2 [148].

The presence of GLO2 activity and that of the enzyme protein were demonstrated in bovine brain microtubules using immunoblotting with antiGLO2 antiserum, even after three cycles of polymerization [149]. Similar results were obtained with calf brain tubulin preparations. After three aggregation/disaggregation cycles, GLO2 activity remained tubulin-associated, while GLO1 activity disappeared after two cycles [150]. The tubulin-associated GLO2 activity was inhibited by 0.5 mM GTP [150]. Employing a mild purification process, GTP-sensitive (coupled) and GTP-insensitive (uncoupled) proteins were gained, but interestingly, both forms bound to GTP/ATP-agarose [150]. Unfortunately, the protein that remained bound to GLO2 has not been identified. Moreover, the S-glutathionylation of tubulin from UACC-62 cells evoked microtubule dysfunction [151]. Both α- and β-tubulin were modified [151].

#### 3.1.3. High-Energy Bond and S-D-Lactoylglutathione as an Energy Currency: Evolutionary Aspects

Over the years, many hypotheses have been formulated to explain the ubiquitous nature of glyoxalases, but none of these could provide a generally acceptable theory as to their role. In the last fifteen years, a series of papers have discussed the function of the MGO pathway by supposing that the pathway itself might have served as an anaplerotic route for the reductive citric acid cycle (Figure 1), and the emergence of energy-rich bonds was deduced from the concept in a plausible manner [152,153,154].

The thioester bonds were suggested to be the ancestors of high-energy ATP [155]. The free energies (ΔG^0^′) liberated during the hydrolysis of the thioester bond of SDL and the phosphoanhydride bond of ATP are as high as −11.24 kcal/mol (−49.23 kJ/mol) and −7.3 kcal/mol (−32 kJ/mol), respectively, providing the opportunity for either ATP synthesis from ADP and inorganic phosphate or driving endergonic reactions linked to SDL breakage [4,130]. The latter may, perhaps, be the reason for and an explanation of why GLO2 is bound to microtubules (vide supra).

#### 3.1.4. Glutathione and S-D-Lactoylglutathione Transport into Mitochondria

Both GLO2 and SDL were detected in mitochondria [31,32,33,34,35,37,38,39,123,156,157]. However, GLO1 is missing from mitochondria [158]. The uptake of SDL in the case of both [glycine-2-^3^H]glutathione labeling and [^14^C]MGO labeling was fast, but mitochondria rapidly lost radioactivity when ^14^C-labelled SDL was used, suggesting an excretion of the product, probably D-lactate or pyruvate, from mitochondria [156]. It is noteworthy that SDL uptake led to an increase in intramitochondrial GSH (mGSH) and proved to be an ATP-independent process [156]. It was concluded that this mechanism served as a supplementary supply of GSH to mitochondria [156]. The mGSH represents 10℃15% of the total cellular GSH pool and is able to cope with oxidative pressure under normal conditions [159,160]. By decreasing below a certain threshold, mGSH loses its protective efficiency against ROS, and lipid peroxidation and protein destruction occur. Thus, in light of all these, a mechanism protecting mitochondria against oxidative stress could be assigned to SDL transport into mitochondria (Figure 1). Thus, the above-mentioned authors, similar to others working with African trypanosomes that have not found GLO1 in mitochondria, simply offer a function for mitochondrial GLO2 as a thioesterase not linked to ketoaldehyde metabolism [156,158].

However, there are several carriers that can transport GSH into mitochondria: among others, mono- and tricarboxylate carriers, glutamate/aspartate carrier and glutamate carriers [161]. The interorgan GSH traffic is also well-documented. The major organ that exports GSH is the liver [162]. Therefore, the above results alone do not clarify why mitochondria would need another very sophisticated uptake mechanism for the maintenance of the mGSH level. 

This doubt is further increased by the fact that when using SDL as a substrate for oxygen consumption in isolated mitochondria from *Arabidopsis thaliana*, the rate of the resulting SDL oxidation depended on whether the mitochondria originated from NH_4_^+^-grown or NO_3_^−^-grown plants [163]. Although the confirmation of this observation with other sources of mitochondria is still anticipated, SDL can be supposed as a substrate for mitochondrial oxidation.

A modest speculation may be appropriate here.

It currently appears that the mitochondrial uptake of SDL serves the purpose of cell survival by several mechanisms. The SDL concentration, as a result of GLO1 activity, is linked to glycolysis, the activity of which strongly varies in the function of cellular needs. In the case of an increased rate of glycolysis, the flux via glyoxalases also increases [25]. Since cGLO2 is the rate-limiting step in the glyoxalase route, SDL transport into the mitochondria bypasses the cGLO2-catalyzed step, thus preventing the cytosolic accumulation of MGO and the subsequent cell damage. The function of mGLO2 would be to split SDL and to liberate GSH, thus increasing the mGSH level and providing D-lactate for terminal oxidation.

#### 3.1.5. Role of S-D-Lactoylglutathione in Potassium Transport and Cellular Defense

Due to its toxicity, the intracellular level of free MGO has to be strictly controlled and maintained low [164].

Two lines of interrelated defense mechanisms against MGO, produced endogenously or added externally, have been identified in *E. coli,* with this field being a subject of intensive research since the 1990s [165]. Protection against this dicarbonyl is performed by detoxification enzymes, mainly by the glyoxalase path producing SDL as an intermediate, and by the activation of the KefB and KefC K^+^ transport systems (potassium efflux system, Kef), activated by GSH adducts, including SDL [166,167]. Though *E. coli* possesses two SDL-regulated systems for the rapid release of K^+^, those are not equally sensitive to SDL, as KefB and KefC are strongly and weakly activated, respectively [168,169]. The difference in their activation by SDL is due to a higher level of energy barrier to open K^+^ channel in KefC in comparison to KefB [167]. At the same time, GSH displays a counter-regulatory role for K^+^ transport [168]. The relationship between K^+^ transport and MGO detoxification, on the one hand, results in a loss of K^+^, while on the other hand, it leads to the decrease in intracellular pH as a result of the function of the K^+^/H^+^ antiport system [90,168,169]. It is, however, to be noted that the intracellular K^+^ level is restored [168]. A similar mechanism has recently been described for *Salmonella typhimurium* [170]. The activity of the Kef-glyoxalase system seems to be related to the capability of these pathogens to affect intracellular infection and virulence [170].

In *B. subtilis*, in which BSH replaces GSH as a LMW thiol, S-lactoyl-BSH has the same function as SDL in *E. coli* [90,170].

In the coordinated protection against MGO, the focus is either on glyoxalases or on Kefs, depending on the intracellular level of SDL that reflects the MGO burden. It is the SDL pool that determines the activity of KefB and in this way influences the degree of intracellular pH decrease that is the most important protection factor against this electrophile [171]. At very low levels of MGO, glyoxalases are able to detoxify the dicarbonyl and maintain a low concentration of SDL [169]. Hence, KefB is relatively inactive, while at higher levels of MGO, SDL may accumulate, as seen for MGO-loaded hepatocytes, which results in the activation of the K^+^ efflux system [104,169].

KefB and KefC are 601- and 620-amino-acid proteins, respectively, and the sequence analysis revealed an about 70% similarity between these proteins, but an obvious difference in substrate specificity was also recognized [165,172]. Specific additional proteins, KefG and KefF, that are related to the quinine oxidoreductase enzyme family, form complexes with KefB and KefC, respectively [167]. 

To be added, the KefC and GLO1 sequence similarity is low [173]. Only over a relatively short region between putative transmembrane helices, M4 and M5, is the sequence similarity high. This region is believed to function as a GSH-recognition motif [173].

It currently appears that the decrease in pH is sufficient to provide some protection to the cells against MGO toxicity [167]. The mechanism of this protection is, however, unclear. Therefore, a modest speculation may be appropriate here. Under conditions where the metabolite concentrations are small, the hydration of methylglyoxal is thermodynamically unfavored in comparison to its interaction with GSH [4]. However, in the case of MGO excess, due to either an external source or an internal overproduction, GSH content decreases on the one hand, while on the other hand, the V_max_ of GLO2 becomes the upper limit of the flux through the glyoxalases [4,104,113,172]. Hence, due to the imbalance in GLOs, the SDL level cannot be maintained low. It increases and activates the KefB and KefC K^+^ transport systems [169,172]. As a result of the function of the K^+^/H^+^ antiport system, the intracellular milieu becomes more acidic, thus enhancing an acid-catalyzed hydration of MGO, as seen for other aldehydes [174]. The formed gem-diol is chemically less reactive than the MGO itself, and in this way, this simple technique hampers the high rate of protein modification by the aldehyde and enhances the survival of cells. Furthermore, SDL is stable in an acidic milieu [6,7]. Therefore, it keeps the ion gate open instead of being non-enzymatically decomposed. This mechanism may be important but is obviously not sufficient, as GSH-deleted mutants (Δgsh) are sensitive to MGO, providing evidence for some, but not total, protection to *E. coli* in the absence of GSH [166].

In mammals, ATP-sensitive K^+^ channels (K^+^_ATP_) are widely distributed in tissue types and intracellular compartments, providing a link between cellular energetics and electrical excitability [175,176]. An intriguing proposal in this regard is that SDL, which is an intermediate of acetone metabolism, can be responsible for the seizure-controlling effect of the ketogenic diet. It would exert its effect by activating K^+^ outflow from the cells in the brain [13,177].

Vascular cell-membrane-harbored K^+^_ATP_s (cmhK^+^_ATP_) display low activity under physiological circumstances, while their activity drastically rises during metabolic stress [176]. Vasodilators and vasoconstrictors activate and inhibit their activities, respectively, thus resulting in the reduction of membrane excitability or depolarization [178]. It was proven that S-glutathionylation inhibited the channel in a concentration-dependent manner, while deglutathionylation restored the activity [178]. For the majority of oxidant sensitivity, the Cys^178^ residue, harbored intracellularly, was responsible [179]. Prolonged exposure to MGO at a concentration of 1mM resulted in the suppression of the current through cmhK^+^_ATP_ and vasoconstriction and, at the same time, also led to mRNA instability [180]. On the contrary, acute exposure to MGO led to activation by augmenting the open probability of cmhK^+^_ATP_ through a noncovalent and reversible interaction [181]. Glibenclamide (K^+^ channel blocking agent) was inhibitory to MGO action in both cases [180,181]. The differential behaviors of cmhK^+^_ATP_ to acute and prolonged MGO treatment highlight different, but yet unknown, mechanisms behind these observations. In another study, glibenclamide was ineffective in the elimination of MGO inhibition [182]. The reason for this difference could be that in the latter case the effect of MGO on noradrenaline-induced contraction was investigated and a concentration of dicarbonyl one order of magnitude lower was applied [182]. For the underlying molecular mechanisms, miR-9a-3p from the screened microRNA databases increased its expression in cultured A10 rat smooth muscle cells exposed to MGO in micromolar concentrations and subsequently downregulated the SUR2B mRNA of cmhK^+^_ATP_. The functional assays in human embryonic kidney 293 cells (HEK293) proved that K^+^ currents were impaired by the miR-9a-3p induced by the MGO treatment [183]. In a recent report, it was shown that MGO at a 10 μM concentration, through the activation of cmhK^+^_ATP_, triggered endothelial cell dysfunction in human aortic endothelial cells by activating the JNK/p38 MAPK pathway [184].

Several types of mitochondrial potassium channels (mitK^+^_ATP_) have been described, with Ca^2+^-activated, ATP-regulated and voltage-gated channels having functional roles in mitochondrial volume, respiration and membrane potential (ΔΨ) regulation [185]. The inward potassium flux decreases ΔΨ stimulation to respiration-modulating ROS production [186,187]. ROS have a double-edged role. They are protective against apoptosis but, at the same time, destructive toward targeted proteins [188,189]. Although there are several target proteins for S-glutathionylation in mitochondria, there is no information so far about the S-glutathionylation of mitK^+^_ATP_, and the effects of either MGO or SDL are not yet elucidated. Since the biophysical features of these channels are similar to those of plasma membrane channels, it is therefore tempting to suggest as a working hypothesis that mitK^+^_ATP_ responds to MGO and SDL similarly to cmhK^+^_ATP_.

## 4. The Role of Extracellular S-D-Lactoylglutathione 

### 4.1. S-D-Lactoylglutathione, Cell Growth and Differentiation

The original observation is traced back to the work on the differentiation of human promyelocytic leukemia HL60 cells and erythroleukemia K562 cells [190]. Since the GLO1/GLO2 activity ratio decreased with the appearance of differentiated cells and GLO2 is known to catalyze the rate-limiting step in the conversion of MGO to D-lactate, it was suggested that immature cells failed to metabolize SDL properly [190]. The N-methylformamide-induced differentiation of HL60 cells to neutrophil-like cells led to a change in GLO’s activities [101] There was not only a decrease in GLO1 activity by 66% compared to controls but the GLO2 activity was also doubled at the same time [101]. The K_M_ values for substrates, however, remained unchanged [101]. The cellular concentrations of both MGO and SDL were significantly lower in differentiated cells than in their non-differentiated counterparts [101]. The rate of D-lactate formation and of glucose consumption increased during differentiation by 75% and 44%, respectively [101].

**Figure 3:**
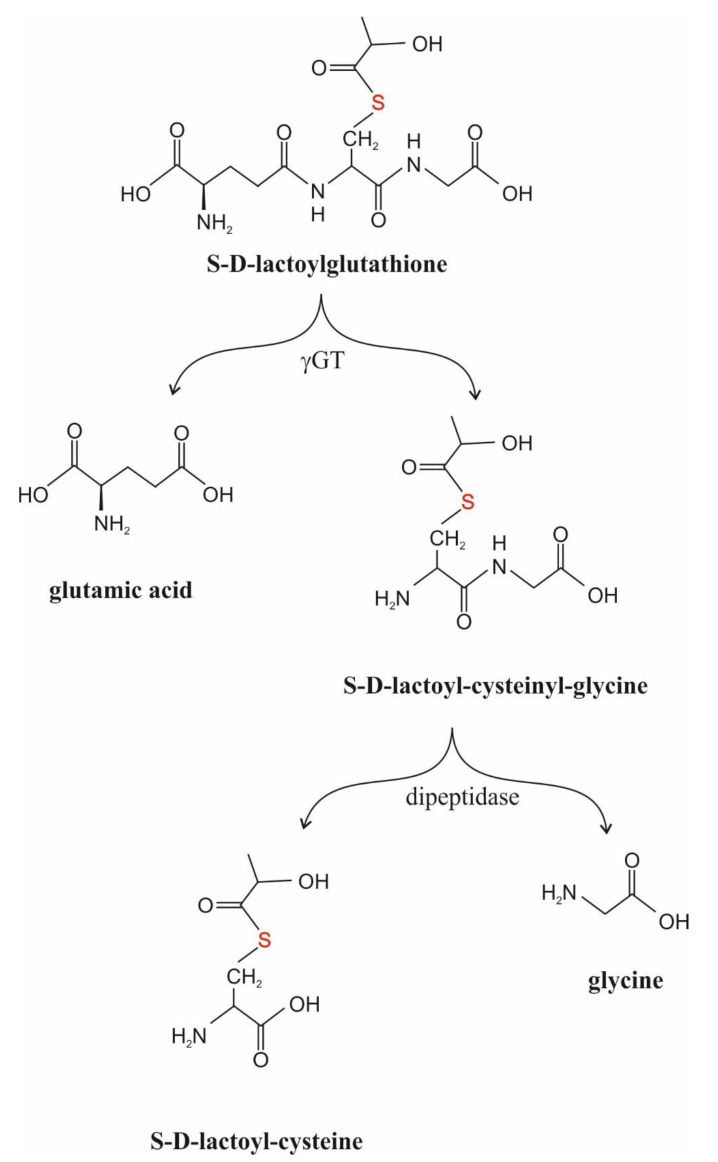
S-D-lactoylglutathione breakdown by γ-glutamyl-transferase and dipeptidase. Abbreviation: γGT, γ-glutamyl-transferase.

When SDL was added to the incubation medium of HL60 cells, a U-shaped profile for the inhibition of cell proliferation and cell viability, as assessed with the Trypan blue dye exclusion test, was obtained [103]. The maximal effect was seen at 500 μM SDL, and the cell proliferation rate and cell viability were only 16% and less than 80% of control values at this concentration, respectively [103]. When the time course of inhibition was investigated in the presence of 500 μM SDL, a sharp decrease in cell viability was observed after 1 day of incubation, while afterwards the growth kinetics of the surviving cells were similar to controls [191]. Interestingly, mature human neutrophils were not affected by the same concentration of SDL [191]. The elevation of the fetal calf serum content of the incubation medium led to a rise in the inhibitory effect of 80 μM SDL on HL60 cell growth [110]. It is to be noted that there was an increase in the percentage of cells in the G_0_–G_1_ phase parallel to a decrease in the number of cells in the G_2_–M phase in the presence of concentrations of SDL higher than 500 μM [102,103]. The activity of GLO1, GLO2 and γGT in SDL-pretreated HL60 cells showed a concentration-dependent increase [103]. The composition of the incubation medium, however, had an effect upon the inhibition of cell proliferation by SDL. The higher the fetal calf serum concentration of the medium, the higher the number of viable cells [108]. These results suggest that SDL influences microtubule assembly during differentiation [102,103]. Externally added SDL was rapidly (within 3 h of culture) consumed without any increase in the intracellular SDL level and decreased DNA synthesis as measured by ^3^H–thymidine incorporation into DNA [103,108,191]. The IC_50_ values for the decrease in cell viability and ^3^H–thymidine incorporation into DNA agreed, showing 66–82 μM and 72–74 μM, respectively [108,191]. As to the mechanism, it remained unknown and is still obscure. However, it is interesting to notice that the half-lives of SDL were about 35 min and 40 min in the presence and in the absence of HL60 cells, respectively [108]. The effect of cells on the phenomenon raises a role for γGT in the events, stressing a role for SDL metabolites rather than SDL itself (Figure 3).

In other laboratories that were influenced by the promine/retine theory of Albert Szent-Györgyi, the relationship between GLO activities and cell differentiation as well as cell division were investigated under different experimental circumstances. The data were, however, inconsistent. There are publications in which the authors suggested a role for GLOs in cell cycle regulation, while others reported on the lack of any involvement in controlling growth [192,193,194,195,196,197,198,199,200]. Unfortunately, neither of the papers reported on SDL. 

### 4.2. S-D-Lactoylglutathione and Secretion

In polymorphonuclear leukocytes, concanavalin A was reported to increase microtubule assembly and, at the same time, activated both GLOs in a dose-dependent manner [201]. The activation of GLO1 was considerably higher than that of GLO2, raising the feasibility of SDL accumulation [201]. The addition of SDL enhanced histamine release from IgE-provoked human leukocytes, while the inhibition of GLO1 activity decreased the release [202]. Its action was believed to develop through microtubule assembly by an unknown mechanism [202]. However, the effect of SDL itself is doubtful and its metabolites may be responsible for the events (see Figure 3). 

**Figure 4:**
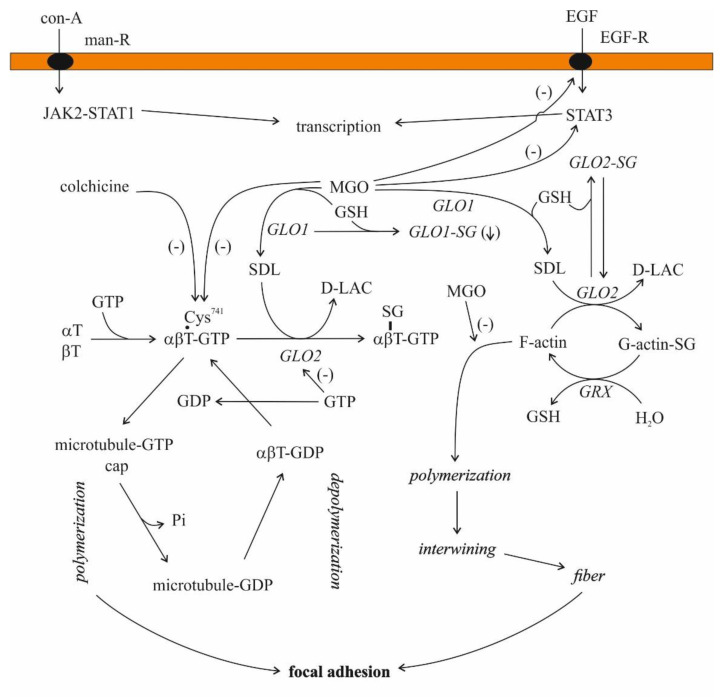
Schematic illustration of mechanisms of the possible participation of GLO2 and SDL in the interactions of microtubules and actin filaments in relation to extracellular stimuli. The data upon which the figure is based are from the literature already cited in text, with the exception of the following articles that are cited here first [204,205,206]. Note, immunological studies proved the association between microtubules and GLO2 [149,150]. The association between actin and GLO2 results in a decrease in the enzymatic activity [128]. As can be seen, S-glutathionylation has an opposite effect on tubulin and actin, and this opposite effect is also recognized in the case of Rho factor, too. Rho promotes both the formation and the contractility of fibers, which are amplified by the positive feedback to it. While it enhances the stabilization of microtubules, there is a negative feedback between microtubule polymerization and Rho [204]. At the same time, fibers activate, while microtubules inactivate focal adhesion [204]. Abbreviations: MGO, methylglyoxal; GSH, reduced glutathione; SDL, S-D-lactoylglutathione; GLO1, glyoxalase I; GLO1-SG, glutathionylated glyoxalase I; GLO2, glyoxalase II; GLO2-SG, glutathionylated glyoxalase II; D-LAC, D-lactate; EGF, epidermal growth factor; EGF-R, EGF receptor; GRX, glutaredoxin; JAK, Janus kinase; man-R, mannose 6-phosphate receptor; STAT, signal transducer and activator of transcription; αT, α-tubulin; βT, β-tubulin; Pi, inorganic phosphate; (-), inhibition; (↓), decrease in activity.

Serum-treated zymosan particles increased GLO1 activity and decreased GLO2 activity in human neutrophils in vitro, and as expected, SDL concentrations increased, parallel to the changes in enzymatic activities, and GSH levels were diminished [69,72]. It was revealed that the changes in the enzymatic activities were due to a non-competitive activation and a non-competitive inhibition of GLO1 and GLO2, respectively [72]. The addition of TPA to human cultured leukocytes led to the same changes in GLO1 and GLO2 activities [72]. A correlation between intracellular SDL concentration and microtubule length was also found. Therefore, the authors speculated on the regulatory role of SDL in microtubule length [72]. On the contrary, the depletion of GSH levels in neutrophiles inhibited microtubule assembly [203]. Hence, it seems likely that GSH and SDL play a counterregulatory role in microtubule assembly (Figure 4). 

Low concentrations of externally added SDL increased granule secretion from TPA-activated neutrophils, while at concentrations higher than 100 μM, the compound inhibited granule secretion [207]. When elaborating the explanation of the results, it should be considered that the addition of TPA to human leukocytes or to yeast, by increasing GLO1 and decreasing GLO2 activities, created a situation for possible intracellular SDL accumulation [68,69,70,71]. Hence, the effects of SDL metabolites, as suggested above for the cases of externally added SDL and intracellularly produced SDL, cannot be distinguished. Therefore, further research is warranted.

A similar shape of the response curve to SDL was gained when the effect of SDL on chemotaxis was investigated [208]. The preincubation time needed for the event to develop was 30 min [207]. It is not yet clear what sort of mechanism may be involved in the event (see above the possible role of SDL metabolites).

Taken together, externally added SDL is to be decomposed in the medium, probably mainly due to the action of enzymes present in the fetal calf serum, and is not taken up by the cells. Rather, its degradation products, either S-D-lactoyl-cysteinyl-glycine or S-D-lactoyl-cysteine, may inhibit DNA synthesis and granule secretion (Figure 3). 

The inability of SDL to cross cell membranes has been reported [29,104]. In addition, glyoxalases are lacking in the extracellular space, as they are exclusively harbored in compartments inside the cells. Despite all these, as previously assumed, SDL has recently been detected in rat as well as human sera [107,209,210,211]. All these raise a series of questions: (i) what the source(s) of extracellular SDL is (are) and what the mechanism for its transport is; (ii) what the reason for earlier negative results is; and (iii) how the findings of previous experiments with externally added SDL should be explained?

## 5. Brief Summary of the Possible Roles of S-D-Lactoylglutathione in Diseases

### 5.1. Hematological Disorders

MGO production strongly hinges upon the generation of triose-phosphates (TPs) [13,212]. Although the measure of the TP pool is not exclusively dependent on the rate of glycolysis, as the Entner–Doudoroff pathway, hexose-monophosphate route and other elements, such as xylitol metabolism or α-glycerophosphate dehydrogenase (E.C.1.1.1.8), also contribute to TP generation, the determinative source of the TP pool is, indeed, glycolysis [213]. One of the TPs, dihydroxyacetone-phosphate (DHAP), is converted to MGO, either enzymatically or non-enzymatically, thus by-passing the second part of glycolysis [212] (Figure 1). 

Glycolytic enzyme deficiencies usually manifest in red blood cells, as their energy production solely relies on anaerobic glucose breakdown. Abnormalities of the Embden–Meyerhof pathway disturb the integrity of red blood cells, thus shortening their life-span and resulting in hemolytic anemia. Hence, any inherited metabolic disorder downstream of DHAP may result in an increase in MGO formation by enhanced flux. 

SDL is unable to cross cell membranes and its production is inevitably bound to the intracellular space, as GLO1 is not present in the plasma. Therefore, it would only be measurable in human and animal plasma when red blood cells were disrupted and SDL leaked from damaged cells [211]. Consequently, hemolysis occurring for whatever reason ought to result in a given level of SDL in the plasma in comparison to healthy controls. A case was already reported for hepatocytes from which SDL release was observed when the cells lost their viability [104,214]. Since red blood cells represent the most abundant cell type in the blood, the falsifying effect of other types of cells does not make sense. Recently, SDL was detected in rat as well as human sera, giving some support to the suggestion but still requiring further corroboration [111,210,211].

Although by far the most frequent abnormality is pyruvate kinase deficiency, the best-characterized enzymopathy is the triose-phosphate isomerase (TPI) deficiency.

TPI catalyzes the conversion of DHAP to glyceraldehyde 3-phosphate (GA3P). The reaction on its own is shifted toward the formation of DHAP, as K′_eq_ = 0.0475, ΔG_o_^′^ = 1.8 kcal/mol (7.88 kJ/mol) and at equilibrium approximately 96% of TPs are in the form of DHAP [215]. Biochemically, a marked decrease in TPI activity leads to DHAP accumulation in red blood cells; even 20- and 60-fold increases are possible [216]. Oddly enough, ATP content does not fall seriously [217].

The link between MGO production and glucose turnover is well-known. However, the methylglyoxal synthase governing the conversion of DHAP to MGO and liberating inorganic phosphate in prokaryotes is not present in multicellular organisms. Thus, the only way for MGO formation and phosphate release from DHAP in mammals is the non-enzymatic reaction [212]. Since a sharp decrease in TPI activity blocks the formation of GA3P from DHAP, leading to the accumulation of the latter, increased concentrations of MGO and its metabolites are expected in this disorder. Until now, the case of a Hungarian family with a TPI deficiency has been the only case being thoroughly studied. Although the SDL content was not measured, the D-lactate levels were elevated about four-fold in blood plasma, suggesting an increased flux through the glyoxalase pathway, with a possible concomitant rise in SDL [218]. 

Although other enzyme deficiencies downstream of TPI may also result in TP accumulation followed by hemolysis, direct evidence for the change in MGO metabolism is still lacking. A semi-clinical observation, however, further substantiates the assumption that the enzyme deficiencies of the second part of the glycolytic path may increase the flux through the glyoxalase route. Red blood cells obtained from non-ketotic type 2 diabetic patients showed different levels of DHAP and MGO when exposed to 30 mM glucose for 32 h, and the rate of MGO formation and glyceraldehyde-3-phosphate dehydrogenase (GAPDH) activity exhibited a strong negative correlation [219,220]. The higher the enzymatic activity, the lower the level of MGO, and this relationship was also seen between red blood cell GAPDH activities and plasma MGO levels normalized to blood glucose in type 1 diabetic patients [219,221].

An additional line of evidence comes from the experiments with human platelets, in which monoiodo-acetamide, an alkylating agent inhibiting GAPDH activity, increased the intracellular DHAP concentration and led to SDL accumulation in thrombin-treated cells [109].

The lack of GLO2 in red blood cells has been reported both in humans and in horses [29,222,223]. Erythrocyte GLO2 deficiency was described in a family in which the members were distinguished as homozygous and heterozygous for GLO2 deficiency [222]. The gene is located on chromosome 16p13, and the deficiency (OMIM 138760) follows an autosomal recessive trait [222]. Interestingly, the leukocyte GLO2 activity was not affected [222]. Elliptocytosis was detected in three of four affected persons, without any other clinical abnormality [222,224]. It was concluded that elliptocytosis and GLO2 deficiency were inherited in an independent manner [222].

Investigating domestic animals, two horses were identified as not having erythrocyte GLO2 [223]. Nonetheless, the absence of GLO2, similarly to humans, was without any clinical significance [223]. GLO2 deficiency was also verified in a 12-year-old horse, but this abnormality did not affect his racing performance [29]. MGO was slowly metabolized by GLO1 in the GLO2-deficient red blood cells, and SDL was converted spontaneously to D-lactate, while it was not transported to the extracellular space [29]. Similar to the human case, leukocytes from the GLO2-deficient horse did not share the GLO2 deficiency; they did perform GLO2 activity [29].

The fact that GLO2 was absent from erythrocytes but was present in leukocytes or, in a broader context, in nuclear cells raised the hypothesis that this discrepancy was due to an unstable variant of the enzyme that was rapidly degraded in the cells but could only be replaced in nucleated cells [29,222]. If this were so, then the absence of GLO2 would only be a pseudo-absence and would address an intriguing question of whether a real GLO2 deficiency could be without any clinical symptom. From this point of view, it is worth noting that *Leishmania braziliensis* seems to lack the GLO2 gene, and there are tumor tissues in which GLO2 activity is obviously missing [225,226]. Finally, like control cells, silencing GLO2 in normal human prostate epithelial cells did not show evidence of cell damage [227]. 

All these together display that the absence of GLO2 in red blood cells, and perhaps in other cells, is not incompatible with life, but this does not necessarily mean that clinical symptoms cannot appear.

### 5.2. Diabetes Mellitus

Red blood cells do not require insulin for glucose uptake, as their main functional glucose transporter, glucose transport protein 1, is not regulated by insulin [228,229]. Hence, in the case of hyperglycemia, the intracellular glucose concentration approaches the ambient extracellular level.

In in vitro cultures of human red blood cells, the effects of elevated glucose concentrations on MGO production and degradation were investigated, and under hyperglycemic conditions (0–100 mM) the increase in the flux of MGO metabolized to D-lactate via the glyoxalase route was almost proportional to the initial glucose concentration and the SDL concentration was elevated by 164% [25]. The size of the TP pool increased in experimental hyperglycemia, and consequently, the formation of MGO was also elevated [230]. The glyoxalase activities in erythrocytes did not differ under normoglycemic and hyperglycemic conditions [25]. These data clearly show that SDL formation in red blood cells is linked to the rate of glycolysis and the plasma glucose level [25].

When HEK293T cells were cultured in a high-glucose medium (25 mM) the addition of metformin did not change the intracellular SDL level. At the same time, insulin resulted in a three-fold increase [231]. Moreover, insulin induced an upregulation of GLO1 and downregulated GLO2 without any change in the total enzymatic activities [231], suggesting a specific activity modulation of glyoxalases by a yet unknown PTPM mechanism. Importantly, the MGO influx was enhanced by insulin, which is in a good agreement with an earlier finding according to which insulin increased glucose formation from MGO in isolated hepatocytes originating from streptozotocin-induced diabetic mice [231,232].

The increase in the levels of MGO and its metabolites in the whole blood and urine of people with diabetes has been corroborated by direct measurements [13,233]. However, there are only a few human data on SDL in diabetics. The convincing finding of these investigations was that SDL levels were increased [105,110]. Yet, there is one study in which the levels of MGO, SDL and D-lactate were determined in the same individuals. The concentration of all three metabolites in blood were higher in people with diabetes than in their healthy counterparts, indicating a higher flux of metabolites through the glyoxalase path during hyperglycemic states [110]. 

Observations of streptozotocin-induced diabetic rats proved that the whole blood SDL concentration was increased in diabetic animals in comparison to controls, while this difference was not seen in the case of mice, despite the marked difference in plasma MGO levels [107,234].

### 5.3. Thromboembolic Disorders

In thrombin-exposed human platelets, SDL accumulation was detected, concomitant with the fall in the GSH level, and this event was time- and dose-dependent [106,109]. Neither GLO1 activity nor GLO2 activity were affected by the thrombin addition [106]. Notably, SDL was under the detection limit in resting platelets [106]. The addition of either TPA or Ca^2+^ ionophore A23187, however, resulted in SDL accumulation in platelets [106]. It seems that the functional activation of platelets results in the accumulation of SDL.

### 5.4. B_1_ Vitamin (Thiamine) Deficiency

It was an old observation that the symptoms of B_1_ avitaminosis and MGO intoxication were similar [235]. There is a very common cause of B_1_ avitaminosis, and this is alcoholism, which affects about one fifth of the European population [236]. The frequently occurring malnutrition in alcohol-dependent people results in a thiamine deficiency that causes such alcoholic complications as Wernicke–Korsakoff syndrome, polyneuropathy and cardiomyopathy [237,238]. The pathophysiology of thiamine deficiency is complex and is not fully understood but has a clear connection to glucose metabolism [239]. In supporting the last note, the supplementation of incubation medium of human erythrocytes with thiamine resulted in a fall in both the TP pool and the MGO level, followed by a suppression of D-lactate formation in experimental hyperglycemia [230]. 

### 5.5. Ketotic States Other Than Diabetes Mellitus

Acetone is also a source of MGO formation. Therefore, its concentration may increase in ketotic states [240]. The states in which this kind of change may occur are starvation, congenital propionic and methylmalonic academia, isopropyl alcohol intoxication, ketotic state(s) in alcoholism and disulfiram treatment [240]. In the case of the Atkins diet, the observed ketosis was accompanied by increased plasma acetol and MGO levels [241]. Unfortunately, SDL was not determined.

### 5.6. Paroxysmal Non-Kinesigenic Dyskinesia

Paroxysmal non-kinesigenic dyskinesia (PNKB) is an autosomal-dominant rare movement disorder (OMIM 118800) belonging to the familial paroxysmal dyskinesias [55,59,242]. The encoding gene is located on the chromosome 2, 2q35 and is transcribed into the PNKD (MR-1) protein with three alternatively spliced forms: long (L), medium (M) and short (S) [59,242]. Three mutations have been reported on, all being in the N-terminal region in the mitochondrial targeting sequence [55,59]. PNKD has an activity toward SDL (vide supra), whereas it is unknown whether the mutated forms of the protein have different activities toward SDL, a feature that might be related to clinical events. Nevertheless, it turned out that *Pnkd* knockout mice presented lower GSH levels in their cortex lysates than their wild-type counterparts [56]. Moreover, it was recently revealed that PNKD may have a regulatory role in neurotransmitter release [59,243], a function that is connected to microtubules.

### 5.7. Seroreactivity against Triose-Phosphate Isomerase

Autoantibodies against glycolytic enzymes, including TPI, enolase, pyruvate kinase and phosphoglycerate mutase, were detected in autoimmune gastrointestinal disorders [244]. The most robust likelihood ratio for a positive test in comparison to controls was seen for enolase, with a ratio of 11, while the likelihood ratio for TPI was about 7 [244]. However, it is not clear whether hemolysis may occur in the case of inflammatory bowel diseases, and if so, if there would be any cause-and-effect relationship between the hematologic symptoms and the autoantibodies against the enzymes of the glycolytic sequence. Antibodies against TPI were also found in an Epstein–Barr viral infection accompanied with hemolysis [244,245]. A purified antibody against TPI induced ^51^Cr release from erythrocytes [245]. Hence, the authors suggested that anti-TPI caused hemolysis, which is an infrequent but serious symptom of infectious mononucleosis [245]. Since TPI deficiency was shown to cause hemolysis (wide supra), it is tempting to say that the insufficient activity of TPI is also the causative factor for hemolysis in this case.

## 6. Conclusions

The ongoing ambition in this report is to investigate the role of SDL in extant metabolism. Since the glyoxalase route is widely distributed in nature, SDL also seems to be instrumental, but its role has not yet been clearly discovered in cell metabolism. Is it a substrate for PTPM, a GSH reservoir or an energy currency or perhaps all three (Figure 1)?

The only indisputable observation in the regard of SDL metabolism is its sole production by GLO1 from HTA, which is formed in the course of an interaction between MGO and GSH [13,14]. All other observations on SDL are currently difficult to arrange in a coherent manner. Even its degradation raises questions. Although GLO2 has traditionally been regarded for a long time as the major, if not the only, SDL-catabolizing enzyme, the activity of γGT and PNKD (MR-1) toward SDL opens the way to look at that differently. Particularly in the light of the silencing of either GLO2 or MR-1 that equally inhibited cell proliferation and migration in human cancerous cells [43,227], a modest speculation upon the feasible involvement of SDL in these processes is tempting.

Cells are not in equilibrium. Thus, they continuously need a flow of energy and matter to maintain their state of high structural and metabolic complexity, even under normal conditions. This is particularly true under stress situations. The cellular stress response is a universal mechanism of extraordinary significance, representing a defense reaction of cells to damage and an attempt to reduce damage and maintain or restore the intracellular milieu [246]. There is a set of events in the course of adaptation to changes, including energy production, movement, changes in cell shape, phagocytosis and secretory processes. One part of the metabolic responses to exogenous stressors is the increase in flux through glycolysis, resulting in energy production and, at the same time, an enhanced MGO formation that is deleterious to cells [21,247,248,249]. The intracellular MGO levels rise in the course of stresses, regardless of their nature, as proven for plants, tumors and normal cells [197,248,250,251,252,253,254,255]. It is important that MGO is formed in a small cytosolic locale, stressing the need for an effective detoxifying mechanism. A series of evidence shows that cells are armed with enzymes with MGO-degrading abilities. One of these is the glyoxalase system [21,249,256]. Therefore, the increased rate of glycolysis leads to a parallel rise in flux through glyoxalases [25]. Another portion of stress responses, however, requires the participation of the cytoskeleton.

The glyoxalase pathway is a stress-related route, as under various stress conditions the activity of GLO1 was particularly increased, as proven in experiments with different species [81,248,253,257,258,259,260,261,262]. In contrast, the changes in GLO2 activities in a stressful situation, if measured, were not so clear. In one case it increased, while in another case decreased [81,254,258,259,260,262]. Though the activation of GLO1 itself would already be satisfactory alone in response to SDL accumulation, regardless of the direction of GLO2 changes, an increase in GLO1/GLO2 was always reported, creating the possibility of an SDL accumulation that was further enhanced by the elevation of intracellular MGO levels [248,254,259,260,262]. When examined, a rise in the SDL level was also shown [254]. The peculiar and recurrent changes in glyoxalase activities (an increase in GLO1 and a decrease in GLO2 activity) in response to stress conditions suggest that the increase in the SDL level in the cytosol could be strictly required to drive the cell adaptation/response to the stress condition itself, possibly through PTPMs and/or the transfer of GSH from the cytosol into mitochondria (Figure 1).

In lieu of relevant data, the question of whether GLO2 deficiency and the consequent increase in SDL concentrations have clinical consequences has remained elusive. According to the only found source, SDL has an anti-inflammatory effect without any toxic side-effects, even when added at dose of 8000 mg/ kg body weight to rodents as the highest applied dose [263]. 

The absence of GLO1 is effectively compensated by aldo-keto reductases to prevent MGO accumulation in mice [264]. Age decreases GLO1 activity and results in MGO accumulation, and the knock-down or inhibition by RNAi of the enzyme reduce the lifespan and elevate MGO levels [265,266,267]. In contrast, the overexpression of GLO1 elevates the lifespan [265,266,267,268]. GLO1-deficient zebrafish survived until adulthood without growth deficits and showed increased tissue MGO concentrations [269]. This is due to the fact that there are other enzymes, mainly AR, capable of handling MGO. In the case of low 1, 2-dicarbonyl concentrations, the interaction of MGO with GSH is preferred to hydration [4]. When MGO is in excess, the 2-oxoaldehyde concentration easily leads to an overburden of the glyoxalase route and the GLO2-catalyzed step becomes rate-limiting. Thus, the SDL level cannot be maintained low [4,104]. In the presence of GSH, MGO is reduced to lactaldehyde by AR at a high rate, while if the intracellular GSH is below normal, the metabolic importance of this enzyme as a compensatory mechanism in the disposal of 1, 2-dicarbonyl exceeds that of the glyoxalase route, and instead of catalyzing lactaldehyde formation, acetol is formed [115,264,270]. To sum up, the vital importance of glyoxalases is not doubtless, though their presence is advantageous in anti-carbonyl defense.

Elevated SDL is a “switch on” signal for the potassium transport system in *E. coli*, functioning as a K^+^/H^+^ antiport and making the intracellular milieu more acidic [168,169]. This is a factor enhancing MGO hydration and diminishes the damaging effect of dicarbonyl, as gem-diol is chemically less reactive than the dicarbonyl itself. Furthermore, SDL is stable in an acidic milieu, Therefore, it keeps the ion gate open without being non-enzymatically decomposed. However, over-acidification is not without any danger to the cells.

**Figure 5:**
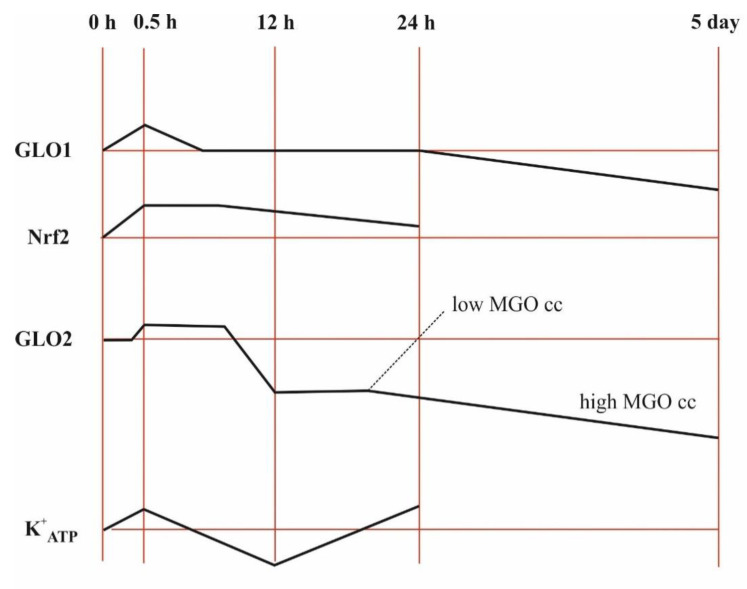
Time course of the changes in glyoxalases, Nrf2 and K^+^_ATP_ in HUVEC. It is to be noted that the GLO1 and GLO2 profiles at day 5 strongly resemble the activities of the enzymes in diabetes mellitus [39,110]. Abbreviations: cc, concentration; MGO, methylglyoxal; GLO1, glyoxalase I; GLO2, glyoxalase II; Nrf2, nuclear factor-erythroid 2 p45 subunit-related factor 2; K^+^_ATP,_ ATP-sensitive potassium channels.

The increase in GLO1/GLO2 in stress indicates that SDL accumulation should be taken into account in stressful situations, and a function may possibly be assigned to that, which is perhaps the interaction with the cytoskeleton. Beside these facts, it is important to recognize that changes after the initiation of cellular stress and insults show time-dependence, determining the change in the concentrations of metabolites over time (Figure 5). 

Referring to the different effects of 0.3 mM and 0.75 mM MGO over time, it is strongly assumed that the degradation of MGO influences events [65]. This may be significant for understanding the acute and chronic clinical effects. In diabetes mellitus, glucose levels are raised, also meaning a stress, so the question emerges of whether the answers should also be similar.

**Figure 6:**
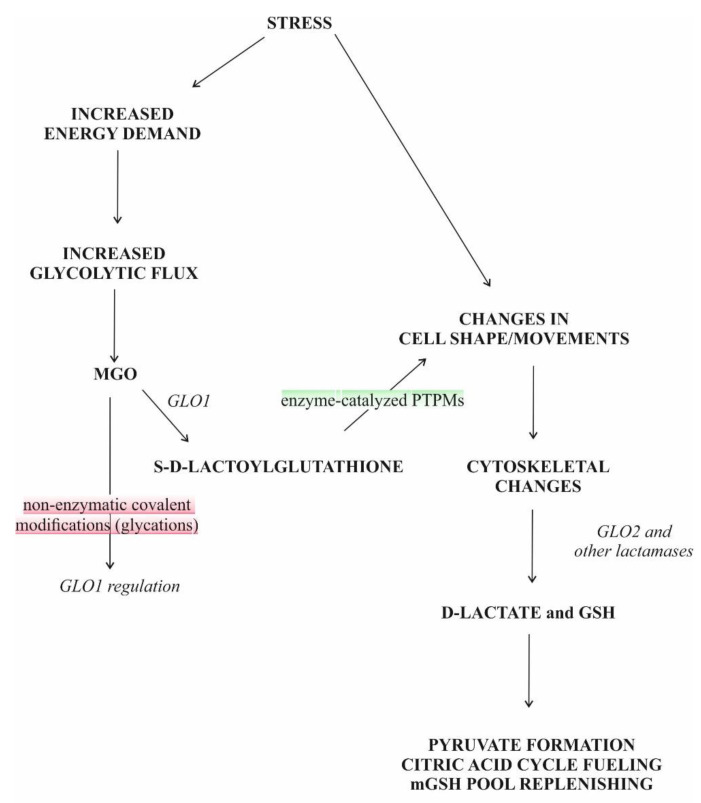
A hypothesis for the role of glyoxalases in the cellular response to stress. Abbreviations: MGO, methylglyoxal; GLO1, glyoxalase I; GLO2, glyoxalase II; GSH, reduced glutathione; mGSH, mitochondrial reduced glutathione; PTPM, post-translational protein modification.

The links among the microfilamental system and glyoxalases as well as SDL are not yet understood. Despite the lack of clear-cut experimental evidence, there is much to suggest again that at least a certain type of connection exists. Although the scientific merit of the suggestion raised may be a subject of debate, it is itself a tempting proposal that such a link might exist under stress situations. The proposal shown in Figure 4 in detailed form is schematically summarized in Figure 6 as a two-stage model.

What we might learn from evolution is that nature does not indulge in luxury. Once a mechanism was found to be useful, similar to a melody that a composer uses again and again, she applies it once again. This melody is the thioester bond in SDL that is widely conserved in biochemical machinery. As noted by Racker, thioester bonds were the ancestors of high-energy ATP, and the free energy liberated during its hydrolysis is higher than that gained when a phosphoanhydride bond is split [155]. Since nature always refines mechanisms, becoming more and more sophisticated, it is not clear why the utilization of the energy conserved in SDL would be wasted away. It is rather likely that the function of it has not yet been recognized. From an evolutionary point of view, this fact has already been stressed [153].

SDL production is a part of MGO metabolism [13,21]. Hence, an elevation of its concentration is expected in all the cases when MGO formation increasingly occurs, as seen in in vitro hyperglycemia [25]. Since the reactions are harbored in the intracellular space, and at least to the present status of knowledge, SDL is unable to cross the cell membrane (vide supra), it is rational to suppose that its extracellular appearance would be a consequence of cell damage [111,209]. From this point of view, the most obvious disorder is hemolysis and, irrespective of missing clinical data, this is a plausible conclusion. At the same time, the detection of an elevated SDL concentration in the plasma would be very difficult. One of the reasons would be the sensitivity of techniques used to measure it, namely, even the elevated concentration would, perhaps, be well below the detection limit. The other reason is that SDL is a substrate for γGT that transfers γ-glutamyl moieties to amino acid or peptide acceptors. Therefore, the presence of its derivatives can rather be expected [49]. Thus, S-D-lactoyl-cysteinyl-glycine or its derivative, S-D-lactoyl-cysteine, may perhaps become detectable in the plasma instead of the parent molecule itself.

Finally, PTPMs by SDL seem to be enzyme-catalyzed, while MGO-induced glycations are non-enzymatic covalent modifications. Since the latter has a lower selectivity, it is prone to accumulate easily and is implicated in disease processes [271]. Therefore, the physiological metabolism-related regulatory role as a linkage between insult and cellular response may, perhaps, rather be assigned to SDL than to MGO. In addition, it might be worth considering two yet-unchecked proposals. On the one hand, there may be a protein-associated SDL-pool in the cytosol [112], and on the other hand, the HTA formed between the protein CYS motifs and MGO may also be a substrate for GLO1, particularly for its phosphorylated form [76]. In this way, S-lact(o)ylation could perhaps also take place in parallel to N-lact(o)ylation, raising intriguing questions.

In the regard of future research, there is a considerable demand for innovative approaches. Perhaps the directions of potential interest could be to seek answers to the following problems, just to name a few, but not to limit to those: 

SDL can have a role in both S-glutathionylation and N-lact(o)ylation. What determines in which reaction it participates? 

MGO formation in mitochondria is a result of non-enzymatic reactions. As seen, GLO1 is not vital for either the cells or mitochondria. Which enzyme supplements GLO1 in the mitochondrion?

No uptake of externally added SDL was detected in reports. How does it act intracellularly? In other words, is there anyway a transport system akin to the mitochondrial SDL uptake system or its metabolites would perform a function? 

The activities of the enzymes of the glyoxalase route vary. In general, the GLO1 activity significantly exceeds that of GLO2 [225,272]. Thus, GLO1/GLO2 creates the possibility of SDL accumulation. Should these warm-up the debate again upon the promine/retine theory of cell division?

However, the duality of GLO2 is also manifested in its role in apoptosis. It has a proapoptotic effect in one tumor, while it is anti-apoptopic in another one [31,43]. If this is the case, then what role does SDL play?

## Abbreviations

AR = aldose reductase, ARE = antioxidant-response element, BSH = bacillithiol, cGLO2 = cytosolic glyoxalase II, cmhK+ATP = cell-membrane-harbored ATP-sensitive potassium channel, DHAP = dihydroxyacetone-phosphate, EGF = epidermal growth factor, γGT = γ-glutamyl-transferase, GA3P = glyceraldehyde 3-phosphate, GAPDH = glyceraldehyde-3-phosphate dehydrogenase, GLO1 = glyoxalase I, GLO2 = glyoxalase II, GLO3 = glyoxalase III, GSH = reduced glutathione, GSNO = S-nitrosoglutathione, HAGH = hydroxyacylglutathione hydrolase, HAGHL = hydroxyacylglutathione hydrolase-like protein, HEK293 = human embryonic kidney 293 cells, HMBLf = human metallo-β-lactamase fold protein, HTA = hemithioacetal adduct, HUVEC = human umbilical venous endothelial cells, K^+^ATP = ATP-sensitive potassium channels, Kef = potassium efflux system, LMW thiol = low-molecular-weight thiol, MAP = microtubule-associated protein, MGO = methylglyoxal, mGSH = intramitochondrial GSH, mitK+ATP = mitochondrial ATP-sensitive potassium channel, mGLO2 = mitochondrial glyoxalase II, MR-1 = myofibrillogenesis regulator 1, NRF2 = nuclear factor-erythroid 2 p45 subunit-related factor 2, PNKD = paroxysmal non-kinesigenic dyskinesia, PTPM = post-translational protein modification, SDL = S-D-lactoylglutathione, sFGH = S-formylglutathione hydrolase, T[SH]2 = trypanothione, TP = triose-phosphate, TPA = phorbol 12-myristate 13-acetate, TPI = triose-phosphate isomerase

## References

[1] Vander Jagt D.L., Dolphin D., Poulson R., Avramovic O. (1989). The glyoxalase system. Glutathione: Chemical, Biochemical and Medical Aspects.

[2] Mannervik B. (2008). Molecular enzymology of the glyoxalase system. Drug Metabol. Drug Interact..

[3] De Bari L., Scirè A., Minnelli C., Cianfruglia L., Kalapos M.P., Armeni T. (2021). Interplay among Oxidative Stress, Methylglyoxal Pathway and S-Glutathionylation. Antioxidants.

[4] Creighton D.J., Migliorini M., Pourmotabbed T., Guha M.K. (1988). Optimization of efficiency in the glyoxalase pathway. Biochemistry.

[5] Reeves M.E., Thornalley P.J. (1993). The hydrolysis of S-D-lactoylglutathione. Biochem. Soc. Transact..

[6] Yamazoye S. (1936). Glyoxalase and its co-enzyme/III. The mechanism of the action of glutathione as the co-enzyme of glyoxalase. J. Biochem..

[7] Racker E. (1951). The mechanism of action of glyoxalase. J. Biol. Chem..

[8] Uotila L. (1981). Thioesters of glutathione. Meth. Enzymol..

[9] Racker E. (1952). Spectrophotometric measurements of the metabolic formation and degradation of thiol esters and enediol compounds. Biochim. Biophys. Acta.

[10] Piskorska D., Jerzykowsky T., Ostrowska M. (1976). Synthesis of S-lactoyl-glutathione using glyoxalase I bound to Sepharose 4B. Experientia.

[11] Kosugi N., Inoue Y., Rhee H.-I., Murata K., Kimura A. (1988). Production of S-lactoylglutathione by glycerol-adapted *Saccharomyces cerevisiae* and genetically engineered *Escherichia coli* cells. Appl. Microbiol. Biotechnol..

[12] Clelland J.D., Thornalley P.J. (1990). Synthesis of ^14^C-labelled methylglyoxal and S-D-lactoylglutathione. J. Label. Comp. Radiopharm..

[13] Thornalley P.J. (1993). The glyoxalase system in health and disease. Mol. Asp. Med..

[14] Kalapos M.P. (1999). Methylglyoxal in living organisms/Chemistry, biochemistry, toxicology and clinical implications. Toxicol. Lett..

[15] Inoue Y., Maeta K., Nomura W. (2011). Glyoxalase system in yeasts: Structure, function, and physiology. Semin. Cell Dev. Biol..

[16] Rae C., Berners-Price S.J., Bulliman B.T., Kuchel P.W. (1990). Kinetic analysis of the human erythrocyte glyoxalase system using ^1^H NMR and computer model. Eur. J. Biochem..

[17] Mannervik B., Ridderström M. (1993). Catalytic and molecular properties of glyoxalase I. Biochem. Soc. Transact..

[18] Creighton D.J., Hamilton D.S. (2001). Brief history of glyoxalase I and what we have learned about metal ion-dependent, enzyme-catalyzed isomerizations. Arch. Biochem. Biophys..

[19] Honek J.F. (2015). Glyoxalase biochemistry. BioMol Concepts.

[20] Kammerscheit X., Hecker A., Rouhier N., Chauvat F., Cassier-Chauvat C. (2020). Methylglyoxal Detoxification Revisited: Role of Glutathione Transferase in Model Cyanobacterium *Synechocystis* sp. Strain PCC 6803. Mol. Biol. Physiol..

[21] Thornalley P.J. (1990). The glyoxalase system: New developments towards functional characterization of a metabolic pathway fundamental to biological life. Biochem. J..

[22] Feierberg I., Cameron A.D., Åqvist J. (1999). Energetics of the proposed rate-determining step of the glyoxalase I reaction. FEBS Lett..

[23] Lages N.F., Cordeiro C., Sousa Silva M., Ponces Freire A., Ferreira A.E.N. (2012). Optimization of time-course experiments for kinetic model discrimination. PLoS ONE.

[24] Sellin S., Mannervik B. (1983). Reversal of the reaction catalyzed by glyoxalase I/Calculation of the equilibrium constant for the enzymatic reaction. J. Biol. Chem..

[25] Thornalley P.J. (1988). Modification of the glyoxalase system in human red blood cells by glucose in vitro. Biochem. J..

[26] Luengo A., Abbott K.L., Davidson S.M., Hosios A.M., Faubert B., Chen S.H., Freinkman E., Zachatias L.G., Mathews T.P., Clish C.B. (2019). Reactive metabolite production is a targetable liability of glycolytic metabolism in lung cancer. Nat. Commun..

[27] Hall S.S., Doweyko A.M., Jordan F. (1978). Glyoxalase I enzyme studies. 4 -General base catalyzed enediol proton transfer rearrangement of methyl- and phenylglyoxalglutathionylhemithiol acetal to L-lactoyl- and S-Mandeloylglutathione followed by hydrolysis. J. Am. Chem. Soc..

[28] Vander Jagt D.L. (1993). Glyoxalase II—Molecular characteristics, kinetics and mechanism. Biochem. Soc. Transact..

[29] Rae C., Board P.G., Kuchel P.W. (1991). Glyoxalase 2 deficiency in the erythrocytes of a horse: 1H NMR studies of enzyme kinetics and transport of S-lactoylglutathione. Arch. Biochem. Biophys..

[30] Ball J.C., Vander Jagt D.L. (1981). S-2-hydroxyacylglutathione hydrolase (glyoxalase II): Active-site mapping of a nonserine thiolesterase. Biochemistry.

[31] Antognelli C., Frosini R., Santolla M.F., Pierce M.J., Talesa V.N. (2019). Oleuropein-induced apoptosis is mediated by mitochondrial glyoxalase 2 in NSCLC A549 cells: A mechanistic inside and possible novel nonenzymatic role for an ancient enzyme. Oxid Med. Cell Longev.

[32] Principato G.B., Rosi G., Talesa V., Giovannini E., Uotila L. (1987). Purification and characterization of two forms of glyoxalase II from the liver and brain of Wistar rats. Biochim. Biophys. Acta.

[33] Talesa V., Uotila L., Koivusalo M., Principato G., Giovannini E., Rosi G. (1988). Demonstration of glyoxalase II in rat liver mitochondria. Partial purification and occurrence in multiple forms. Biochim. Biophys. Acta.

[34] Talesa V., Rosi G., Contenti S., Mangiabene C., Lupatelli M., Norton S.J., Giovannini E., Principato G.B. (1990). Presence of glyoxalase II in mitochondria from Spinach leaves: Comparison with the enzyme from cytosol. Biochem. Int..

[35] Jassem W., Ciarimboli C., Cerioni P.N., Saba V., Norton S.J., Principato G. (1996). Glyoxalase II and glutathione levels in rat liver mitochondria during cold storage in Euro-Collins and University of Wisconsin solutions. Transplantation.

[36] Bito A., Haider M., Handler I., Breitenbach M. (1997). Purification and phenotypic analaysis of two glyoxalase II coding genes from *Saccharomyces cerevisiae,* GLO2 and GLO4, and intracellular localization of the corresponding proteins. J. Biol. Chem..

[37] Maiti M.K., Krishnasamy S., Owen H.A., Makaroff C.A. (1997). Molecular characterization of glyoxalase II from *Arabidopsis thaliana*. Plant Mol. Biol..

[38] Bito A., Haider M., Briza P., Strasser P., Breitenbach M. (1999). Heterologous expression, purification, and kinetic comparison of the cytoplasmic and mitochondrial glyoxalase II enzymes, Glo2p and Glo4p, from *Saccharomyces cerevisiae*. Protein Expr..

[39] Cordell P.A., Futers T.S., Grant P.J., Pease R.J. (2004). The human hydroxyglutathione hydrolase (HAGH) gene encodes both cytosolic and mitochondrial forms of glyoxalase II. J. Biol. Chem..

[40] Saxena M., Bisht R., Roy S.D., Sopory S.K., Bhalla-Sarin N. (2005). Cloning and characterization of a mitochondrial glyoxalase II from *Brassica juncea* that is upregulated by NaCl, Zn, and ABA. Biochem. Biophys. Res. Commun..

[41] Xue M.-Z., Rabbani N., Momiji H., Imbasi P., Anwar M.M., Kitteringham N., Park B.K., Souma T., Moriguchi T., Yamamoto M. (2012). Transcriptional control of glyoxalase 1 by Nrf2 provides a stress-responsive defence against dicarbonyl glycation. Biochem. J..

[42] Urscher M., Alisch R., Deponte M. (2011). The glyoxalase system of malaria parasites—Implications for cell biology and general glyoxalase research. Semin. Cell Dev. Biol..

[43] Antognelli C., Ferri I., Bellezza G., Siccu P., Love H.D., Talesa V.N., Sidoni A. (2017). Glyoxalase 2 drives tumorigenesis in human prostate cells in a mechanism involving androgen receptor and p53-p21 axis. Mol. Carcinog..

[44] Uotila L., Koivusalo M. (1974). Purification and properties of S-formylglutathione hydrolase from human liver. J. Biol. Chem..

[45] Gonzalez C.F., Proudfoot M., Brown G., Korniyenko Y., Mori H., Savchenko A.V., Yakunin A.F. (2006). Molecular basis of formaldehyde detoxification/Characterization of two S-formylglutathione hydrolases from Escherichia coli, FrmB and YeiG. J. Biol. Chem..

[46] Van Straaten K.E., Gonzalez C.F., Valladares R.B., Xu X., Savchenko A.V., Sanders D.A.R. (2009). The structure of S-formylglutathione hydrolase from Agrobacterium tumefaciens. Protein Sci..

[47] Apeshiotis F., Bender K. (1986). Evidence that S-formylglutathione hydrolase and esterase-D polymorphisms are identical. Hum. Genet..

[48] Eiberg H., Mohr J. (1986). Identity of the polymorphisms for esterase-D and S-formylglutathione hydrolase in red-blood-cells. Hum. Genet..

[49] Tate S.S. (1975). Interaction of γ-glutamyl transpeptidase with S-acyl derivatives of glutathione. FEBS Lett..

[50] Meister A., Tate S.S., Griffith O.W. (1981). γ-glutamyltransferase. Meth. Enzymol..

[51] Thornalley P.J. (1998). Glutathione-dependent detoxification of α-oxoaldehydes by glyoxalase system: Involvement in disease mechanisms and antiproliferative activity of glyoxalase I inhibitors. Chem. Biol. Interact..

[52] Whitfield J.B. (2001). Gamma Glutamyl Transferase. Crit. Rev. Clin. Lab. Sci..

[53] Pompella A., De Tata V., Paolicchi A., Zunino F. (2005). Expression of γ-glutamyltransferase in cancer cells and its significance in drug resistance. Biochem. Pharmacol..

[54] Martins A.M., Cordeiro C., Freire A.P. (1999). Glyoxalase II in *Saccharomyces cerevisiae*: In situ kinetics using the 5,5′-dithiobis(2-nitrobenzoic acid) assay. Arch. Biochem. Biophys..

[55] Lee H.-Y., Xu Y., Huang Y., Ahn A.H., Auburger G.W.J., Pandolfo M., Kwiecinski H., Grimes D.A., Lang A.E., Nielsen J.E. (2004). The gene for paroxysmal non-kinesigenic dyskinesia encodes an enzyme in a stress response pathway. Hum. Mol. Genet..

[56] Shen Y., Lee H.-Y., Rawson J., Ojha S., Babbitt P., Fu Y.-H., Ptáĉek L.J. (2011). Mutations in PNKD causing paroxysmal dyskinesia alters protein cleavage and stability. Hum. Mol. Genet..

[57] Pettinati I., Brem J., Lee S.Y., McHugh P.J., Schofield C.J. (2016). The chemical biology of human metallo-β-lactamase fold proteins. Trends Biochem..

[58] Erro R., Bhatia K.P., Espay A.J., Striano P. (2017). The epileptic and non-epileptic spectrum of paroxysmal dyskinesias: Channelopathies, synaptopathies, and transportopathies. Mov. Disord..

[59] Ghezzi D., Viscomi C., Ferlini A., Gialandi F., Mereghetti P., DeGrandis D., Zeviani M. (2009). Paroxysmal non-kinesigenic dyskinesia is caused by mutations of the MR-1 mitochondrial targeting sequence. Hum. Mol. Genet..

[60] Gong Y., He H., Liu H., Zhang C., Zhao W., Shao R. (2014). Phosphorylation of myofibrillogenesis regulator-1 activates the MAPK signaling pathway and induces proliferation and migration in human breast cancer MCF7 cells. FEBS Lett..

[61] Birkenmeier G., Stegemann C., Hoffmann R., Günther R., Huse K., Birkemeyer C. (2010). Posttranslational modification of human glyoxalase 1 indicates redox-dependent regulation. PLoS ONE.

[62] Antognelli C., Talesa V.N. (2018). Glyoxalases in urological malignancies. Int. J. Mol. Sci..

[63] Singla-Pareek S.L., Kaur C., Kumar B., Pareek A., Sopory S.K. (2020). Reassessing plant glyoxalases: Large family and expanding functions. New Phytol..

[64] Morgenstern J., Campos M., Nawroth P., Fleming T. (2020). The glyoxalase system—New insights into an ancient metabolism. Antioxidants.

[65] Dafre A.L., Goldberg J., Wang T., Spiegel D.A., Maher P. (2015). Methylglyoxal, the foe and friend of glyoxalase and Trx/TrxR systems in HT22 nerve cells. Free Radic. Biol. Med..

[66] Xu Y., Chen X. (2006). Glyoxalase II, a detoxifying enzyme of glycolysis byproduct methylglyoxal and a target of p63 and p73, is a pro-survival factor of the p53 family. J. Biol. Chem..

[67] Anaki N., Morimasa T., Salai T., Tokuoh H., Yunoue S., Kamo M., Miyazaki K., Abe K., Saya H., Tsugita A. (2000). Comparative analysis of brain proteins from p53-deficient mice by two-dimensional electrophoresis. Electrophoresis.

[68] Gillespie E. (1981). The tumor promoting phorbol diester, 12-O-tetradecanoylphorbol-13-acetate (TPA) increases glyoxalase I and decreases glyoxalase II in human polymorphonuclear leukocytes. Biochem. Biophys. Res. Commun..

[69] Thornalley P.J., Bellavite P. (1987). Modification of the glyoxalase system during functional activation of human neutrophils. Biochim. Biophys. Acta.

[70] Murata K., Sato N., Inoue Y., Kimura A. (1989). S-D-lactoylglutathione: Control of the cellular level by a yeast glyoxalase system. Agric. Biol. Chem..

[71] Murata K., Inoue Y., Rhee H., Kimura A. (1989). 2-oxoaldehyde metabolism in microorganisms. Can. J. Microbiol..

[72] Thornalley P.J., Della Bianca V., Bellavite P., Rossi F. (1987). S-D-lactoylglutathione in resting and activated human neutrophils. Biochem. Biophys. Res. Commun..

[73] Inoue Y., Choi B.-Y., Murata K., Kimura A. (1989). Sexual response in *Saccharomyces cerevisiae*: Alteration of enzyme activity in the glyoxalase system by mating factor. Biochem. Biophys. Res. Commun..

[74] Inoue Y., Choi B.-Y., Murata K., Kimura A. (1990). Sexual response of Saccharomyces cerevisiae: Phosphorylation of yeast glyoxalase I by a cell extract of mating factor-treated cells. J. Biochem..

[75] Kimura A., Inoue Y. (1993). Glyoxalase I in microorganisms: Molecular characteristics, genetics and biochemical regulation. Biochem. Soc. Transact..

[76] Van Herreweghe F., Mao J., Chaplen F.W.R., Grooten J., Gevaert K., Vandekerckhove J., Vancompernolle K. (2002). Tumor Necrosis Factor-Induced (cytokine) Modulation of Glyoxalase I Activities Through Phosphorylation by PKA Results in Cell Death and Is Accompanied by the Formation of a Specific Methylglyoxal-Derived AGE. Proc. Natl. Acad. Sci. USA.

[77] De Hemptine V., Rondas D., Toepoel M., Vancompernolle K. (2007). Tumour necrosis factor induces phosphorylation primarily of the nitric-oxide-responsive form of glyoxalase I. Biochem. J..

[78] De Hemptine V., Rondas D., Toepoel M., Vancompernolle K. (2009). Phosphorylation on Thr-106 and NO-modification of glyoxalase I suppress the TNF-induced transcriptional activity of NF-κB. Mol. Cell Biochem..

[79] Teijero J.M., Marini P.E. (2020). Hormone-regulated PKA activity in porcine oviductal epithelial cells. Cell Tissue Res..

[80] Mitsumoto A., Kim K.-R., Oshima G., Kunimoto M., Okawa K., Iwamatsu A., Nakagawa Y. (1999). Glyoxalase I is a novel nitric-oxide-responsive protein. Biochem. J..

[81] Hasanuzzaman M., Nahar K., Alam M.M., Fujita M. (2012). Exogenous nitric oxide alleviates high temperature induced oxidative stress in wheat (*Triticum aestivum* L.) seedlings by modulating the antioxidant defense and glyoxalase system. Aust. J. Crop. Sci..

[82] Hasanuzzaman M., Nahar K., Hossain M.S., Islam A., Parvin K., Fujita M. (2017). Nitric oxide pretreatment enhances antioxidant defense and glyoxalase systems to confer PEG-induced oxidative stress in rapeseed. J. Plant Interact..

[83] Kehr S., Jortzik E., Delahunty C., Yates J.R., Rahifs S., Becker K. (2011). Protein S-glutathionylation in malaria parasites. Antioxid. Redox Signal..

[84] Melchers J., Dirdjaja N., Ruppert T., Krauth-Siegel R.L. (2007). Glutathionylation of trypanosomal thiol redox proteins. J. Biol. Chem..

[85] Müller S., Liebau E., Walter R.D., Krauth-Siegel E.L. (2003). Thiol-based redox metabolism of protozoan parasites. Trends Parasitol..

[86] Wyllie S., Fairlamb A.H. (2011). Methylglyoxal metabolism… in trypanosomes and leishmania. Semin. Cell Dev. Biol..

[87] Brophy P.M., Crowley P., Barrett J. (1990). Relative distribution of glutathione transferase, glyoxalase I and glyoxalase II in helminthes. Int. J. Parasitol..

[88] Greig N., Wyllie S., Patterson S., Fairlamb A.H. (2009). A comparative study of methylglyoxal metabolism in trypanosomatids. FEBS J..

[89] Sharma S.V., Arbach M., Roberts A., Macdonald C.J., Groom M., Hamilton C.J. (2013). Biophysical features of bacillithiol, the glutathione surrogate of *Bacillus subtilis* and other Firmucites. Chembiochem.

[90] Ferguson G.P., McLaggan D., Booth I.R. (1995). Potassium channel activation by glutathione-S-conjugates in *Escherichia coli*: Protection against methylglyoxal is mediated by cytoplasmic acidification. Mol. Microbiol.

[91] Chandrangsu P., Dusi R., Hamilton C.J., Helmann J.D. (2014). Methylglyoxal resistance in *Bacillus subtilis*: Contributions of bacillithiol-dependent and independent pathways. Mol. Microbiol..

[92] Loi V.V., Rossius M., Antelmann H. (2015). Redox regulation by reversible protein S-thiolation in bacteria. Front. Microbiol..

[93] Suttisansanee U., Honek J.F. (2019). Preliminary characterization of a Ni^2+^-activated and mycothiol-dependent glyoxalase I enzyme from *Streptomyces coelicolor*. Inorganics.

[94] Lee J., Song J., Kwon K., Jang S., Kim C., Baek K., Kim J., Park C. (2012). Human DJ-1 and its analogs are novel glyoxalases. Hum. Mol. Genet..

[95] Richarme G., Mihoub M., Dairou J., Bui L.C., Leger T., Lamouri A. (2015). Parkinsonism-associated protein DJ-1/Park7 is a major protein deglycase that repairs methylglyoxal- and glyoxal-glycated cysteine, arginine, and lysine residues. J. Biol. Chem..

[96] Galligan J.J., Wepy J.A., Streeter M.D., Kingsley P.J., Mitchener M.M., Wauchope O.R., Beavers W.N., Rose K.L., Wang T., Spiegel D.A. (2018). Methylglyoxal-derived posttranslational arginine modifications are abundant histone marks. Proc. Natl. Acad. Sci. USA.

[97] Matsuda N., Kimura M., Queliconi B.B., Kojima W., Mishima M., Takagi K., Koyano F., Yamono K., Mizushima T., Ito Y. (2017). Parkinson’s disease-related DJ-1 functions in thiol quality control against aldehyde attack in vitro. Sci. Rep..

[98] Klotzsch H., Bergmeyer H.-U., Bergmeyer H.U. (1963). Methylglyoxal. and Glutathione. Methods in Enzymatic Analysis.

[99] Warholm M., Guthenberg C., von Bahr C., Mannervik B. (1985). Glutathione transferases from human liver. Meth. Enzymol.

[100] Thornalley P.J., Hooper N.I., Jennings P.E., Florkowski C.M., Jones A.F., Lunec J., Barnett A.H. (1989). The human red blood cell glyoxalase system in diabetes mellitus. Diabetes Res. Clin. Pract..

[101] Hooper N.I., Tisdale M.J., Thornalley P.J. (1988). Modification of the glyoxalase system in human HL60 promyelocytic leukaemia cell during differentiation to neutrophils in vitro. Biochim. Biophys. Acta.

[102] Hooper N.I., Tisdale M.J., Thornalley P.J. (1988). Glyoxalase activity and cell proliferation in Burkitt’s lymphoma and transformed lymphoblast cells in vitro. Cell Mol. Biol..

[103] Thornalley P.J., Tisdale M.J. (1988). Inhibition of proliferation of human promyelocytic leukaemia HL60 cells by S-D-lactoylglutathione in vitro. Leuk. Res..

[104] Kalapos M.P., Garzó T., Antoni F., Mandl J. (1992). Accumulation of S-D-lactoylglutathione and transient decrease of glutathione level caused by methylglyoxal load in isolated hepatocytes. Biochim. Biophys. Acta.

[105] McLellan A.C., Phillips S.A., Thornalley P.J. (1993). The assay of S-D-lactoylglutathione in biological systems. Anal. Biochem..

[106] Leoncini G., Buzzi E., Aprile B. (1993). S-D-lactoylglutathione accumulation in activated human platelets. Int. J. Biochem..

[107] Uchino E., Fukushima T., Tsunoda M., Santa T., Imai K. (2004). Determination of rat blood S-D-lactoylglutathione by a column-switching high-performance liquid chromatography with a precolumn fluorescence derivatization with 4-fluoro-7-nitro-2,1,3-benzoxadiazole. Anal. Biochem..

[108] Edwards L., Clelland J.D., Thornalley P.J. (1993). Characteristics of the inhibition of human promyelocytic leukaemia HL60 cell growth by S-D-lactoylgluthathione in vitro. Leuk. Res..

[109] Leoncini G., Buzzi E. (1994). Thrombin induces S-D-lactoylglutathione accumulation by enhancing platelet glycolytic pathway. Int. J. Biochem..

[110] McLellan A.C., Thornalley P.J., Benn J., Sonksen P. (1994). Glyoxalase system in clinical diabetes mellitus and correlation with diabetic complications. Clin. Sci..

[111] Wu J.-X., Zheng H., Yao X., Liu X.-W., Zhu H.-J., Yin C.-L., Liu X., Mo Y.-Y., Huang H.-M., Cheng B. (2019). Comparative analysis of the compatibility effects of Danggui-Sini Decoction on a blood stasis syndrome rat model using untargeted metabolomics. J. Chromatogr..

[112] Rabbani N., Xue M., Thornalley P.J. (2014). Activity, regulation, copy number and function int he glyoxalase system. Biochem. Soc. Transact..

[113] Shin M.J., Edinger J.W., Creighton D.J. (1997). Diffusion-dependent kinetic properties of glyoxalase I and estimates of the steady-state concentrations of glyoxalase pathway intermediates in glycolyzing erythrocytes. Eur. J. Biochem..

[114] Thornalley P.J. (2003). Glyoxalase I—Structure, function and a critical role in the enzymatic defence against glycation. Biochem. Soc. Transact..

[115] Vander Jagt D.L., Hassenbrook R.K., Hunsaker L.A., Brown W.M., Royer R.E. (2001). Metabolism of the 2-oxoaldehyde methylglyoxal by aldose reductase and by glyoxalase-I: Roles for glutathione in both enzymes and implications for diabetic complications. Chem. Biol. Interact..

[116] Kalapos M.P., Wu L. (2014). Can methylglyoxal be oxidized to CO2 in vascular smooth muscle cells?. J. Investig. Biochem..

[117] Knorre D.G., Kudryashova N.V., Godovokova T.S. (2009). Chemical and functional aspects of posttranslational modification of proteins. Acta Nat..

[118] Grek C.L., Zhang J., Manevich Y., Townsend D.M., Tew K.D. (2013). Causes and consequences of cysteine S-glutathionylation. J. Biol. Chem..

[119] Johnstone V.P.A., Hool L.C. (2014). Glutathionylation of the L-type Ca^2+^ channel in oxidative stress-induced pathology in the hearth. Int. J. Mol. Sci..

[120] Mailloux R.J., Willmore W.G. (2014). S-glutathionylation reactions in mitochondrial function and disease. Front. Cell Dev. Biol..

[121] Yang Y., Jin X., Jiang C. (2014). S-glutathionylation of ion channels: Insights into the regulation of channel functions, thiol modification crosstalk, and mechanosensing. Antioxid. Redox Signal..

[122] Wilson C., González-Billault C. (2015). Regulation of cytoskeletal dynamics by redox signaling and oxidative stress: Implications for neuronal development and trafficking. Front. Cell Neurosci..

[123] Sciré A., Tanfani F., Saccucci F., Bertoli E., Principato C. (2000). Specific interaction of cytosolic and mitochondrial glyoxalase II with acidic phospholipids in form of liposomes results in the inhibition of the cytosolic enzyme only. Proteins.

[124] Mailloux R.J. (2020). Protein S-glutathionylation reactions as a global inhibitor of cell metabolism for the desensitization of hydrogen peroxide signals. Redox Biol..

[125] Cooper A.J.L., Pinto J.T., Callery P.S. (2011). Reversible and irreversible protein glutathionylation: Biological and clinical aspects. Expert Opin. Drug Metab. Toxicol..

[126] Cianfruglia L., Galeazzi R., Massaccesi L., Spaccini R., Caniglia M.L., Principato G., Armeni T. (2014). Glyoxalase II promotes “in vitro” S-glutathionylation. Free Radic. Biol. Med..

[127] Dominko K., Ðikic D. (2018). Glutathionylation: A regulatory role of glutathione in physiological processes. Arch. Hig. Rada Toksikol..

[128] Ercolani L., Sciré A., Galeazzi R., Massaccesi L., Cianfruglia L., Amici A., Piva F., Urbanelli L., Emilliani C., Principato G. (2016). A possible S-glutathionylation of specific proteins by glyoxalase II: An in vitro and in silico study. Cell Biochem. Funct..

[129] Galeazzi R., Laudadio E., Falconi E., Massaccesi L., Ercolani L., Mobbili G., Minnelli C., Sciré A., Cianfruglia L., Armeni T. (2018). Protein-protein interactions of human glyoxalase II: Findings of a reliable docking protocol. Org. Biomol. Chem..

[130] Darnell J., Lodish H., Baltimore D. (1986). Molecular Cell Biology.

[131] Tang D.D., Gerlach B.D. (2017). The roles and regulation of actin cytoskeleton, intermediate filaments and microtubules in smooth muscle cell migration. Respir. Res..

[132] Wang J., Boja E.S., Tan W., Tekle E., Fales H.M., English S., Mieyal J.J., Chock P.B. (2001). Reversible glutathionylation regulates actin polymerization in A431 cells. J. Biol. Chem..

[133] Sakai J., Li J., Subramanian K.K., Mondal S., Bajrami B., Hattori H., Jia Y., Dickinson B.C., Zhong J., Ye K. (2012). Reactive oxygen species-induced actin glutathionylation controls actin dynamics in neutrophils. Immunity.

[134] James A.M., Hoogewijs K., Logan A., Hall A.R., Ding S., Fearnley I.M., Murphy M.P. (2017). Non-enzymatic N-acetylation of lysine residues by acetyl-CoA often occurs via a proximal S-acetylated thiol intermediate sensitive to glyoxalase II. Cell Rep..

[135] Chen A.-N., Luo Y., Yang Y.-H., Fu J.-T., Geng X.-M., Shi J.-P., Yang J. (2021). Lactylation, a Novel Metabolic Reprogramming Code: Current Status and Prospects. Front. Immunol..

[136] Gaffney D.O., Jennings E.O., Anderson C.C., Marentette J.O., Shi T., Schou Oxvig A.-M., Streeter M.D., Johannsen M., Spiegel D.A., Chapman E. (2020). Non-enzymatic lysine lactoylation of glycolytic enzymes. Cell Chem. Biol..

[137] Zhang D., Tang Z., Huang H., Zhou G., Cui C., Weng Y., Liu W., Kim S., Lee S., Perez-Neut M. (2019). Metabolic regulation of gene expression by histone lactylation. Nature.

[138] Khadka S., Barekatain Y., Muller F.L. (2020). Re-Evaluating the Mechanism of Histone Lactylation. https://www.researchgate.net/publication/341582046.

[139] Kulkarni C.A., Brookes P.S. (2019). Many Routes from Glycolysis to Histone PTMs. 2020. Nature “Matters Arising” response to: Zhang et al. Metabolic regulation of gene expression by histone lactylation. Nature.

[140] Dehmelt L., Halpain S. (2004). The MAP2/Tau family of microtubule-associated proteins. Genome Biol..

[141] Conde C., Cóceres A. (2009). Microtubule assembly, organization and dynamics in axons and dendrites. Nat. Rev. Neurosci..

[142] Macconi R.B., Vera J.C., Slebe J.C. (1981). Arginyl residues involvement in the microtubule assambly. Arch. Biochem. Biophys..

[143] Kalapos M.P. (1994). Methylglyoxal toxicity in mammals. Toxicol. Lett..

[144] Miglietta A., Gabriel L. (1986). Methylglyoxal-tubulin interaction: Studies on the aldehyde effects on hepatoma, liver and purified microtubular protein. Res. Commun. Chem. Pathol. Pharmacol..

[145] Fésűs L., Muszbek L., Laki K. (1981). The effect of methylglyoxal on actin. Biochem. Biophys. Res. Commun..

[146] Dianzani M.U. (1979). Biological activity of methylglyoxal and related aldehydes. Submolecular Biology and Cancer.

[147] Gillespie E. (1975). Cell-free microtubule assembly: Evidence for control by glyoxalase. Fed. Proc..

[148] Clelland J.D., Thornalley P.J. (1993). The potentiation of GTP-dependent assembly of microtubules by S-D-lactoylglutathione. Biochem. Soc. Transact..

[149] Di Simplicio P., Vignani R., Talesa V., Principato G. (1990). Evidence of glyoxalase II activity associated with microtubule polymerization in bovine brain. Pharmacol. Res..

[150] Norton S.J., Elia A.C., Chyan M.K., Gillis G., Frenzel C., Principato G.B. (1993). Inhibitors and inhibition studies on mammalian glyoxalase II activity. Biochem. Soc. Transact..

[151] Chen W., Seefeldt T., Young A., Zhan X., Zhao Y., Ruffolo J., Kaushik R.S., Guan X. (2012). Microtubule S-glutathionylation as a potential approach for antimitotic agents. BMC Cancer.

[152] Kalapos M.P. (1997). Possible evolutionary role of methylglyoxalase pathway/Anaplerotic route for surface metabolists. J. Theor. Biol..

[153] Kalapos M.P. (1998). From mineral support to enzymatic catalysis/Further assumptions for the evolutionary history of glyoxalase system. J. Theor. Biol..

[154] Kalapos M.P. (2002). A theoretical approach to the link between oxido-reductions and pyrite formation in the early stage of evolution. Biochim. Biophys. Acta.

[155] Racker E. (1965). Mechanisms in Bioenergetics.

[156] Armeni T., Cianfruglia L., Piva F., Urbanelli L., Cinaglia M.L., Pugnaloni A., Principato G. (2014). S-D-lactoylglutathione can be an alternative supply of mitochondrial glutathione. Free Radic. Biol. Med..

[157] De Bari L., Atlante A., Armeni T., Kalapos M.P. (2019). Synthesis and metabolism of methylglyoxal, S-D-lactoylglutathione and D-lactate in cancer and Alzheimer’s disease. Exploring the crossroad of eternal youth and premature aging. Ageing Res. Rev..

[158] Wendler A., Irsch T., Rabbani N., Thornalley P.J., Krauth-Siegel R.L. (2009). Glyoxalase II does not support methylglyoxal detoxification but serves as a general trypanothione thioesterase in African Trypanosomes. Mol. Biochem. Parasitol..

[159] Marí M., Morales A., Colell A., García-Ruiz C., Fernández-Checa J.C. (2009). Mitochondrial glutathione, a key survival antioxidant. Antioxid. Redox Signal..

[160] Mailloux R. (2018). Mitochondrial antioxidants and the maintenance of cellular hydrogen peroxide levels. Oxid. Med. Cell Longev..

[161] Passarella S., Atlante A., Valenti D., de Bari L. (2003). The role of mitochondrial transport in energy metabolism. Mitochondrion.

[162] Meister A. (1988). Glutathione metabolism and its s.selective modification. J. Biol. Chem..

[163] Borysiuk K., Ostaszewska-Gugajska M., Vaultier M.-N., Hasenfratz-Sauder M.-P., Szal B. (2018). Enhanced formation of methylglyoxla-derived advanced glycation end products in *Arabidopsis* under ammonium nutrition. Front. Plant Sci..

[164] Chaplen F.W.R., Fahl W.E., Cameron D.C. (1996). Method for determination of free intracellular and extracellular methylglyoxal in animal cells grown in culture. Anal. Biochem..

[165] Ferguson G.P. (1999). Protective mechanisms against toxic electrophiles in *Escherichia coli*. Trends Microbiol..

[166] Ferguson G.P., Booth I.R. (1998). Importance of glutathione for growth and survival of *Escherichia coli* cells: Detoxification of methylglyoxal and maintenance of intracellular K^+^. J. Bact..

[167] Booth I.R., Ferguson G.P., Miller S., Li C., Gunasekera B., Kinghorn S. (2003). Bacterial production of methylglyoxal: A survival strategy or death by misadventure?. Biochem. Soc. Transact..

[168] Ferguson G.P., Munor A.W., Douglas R.M., McLaggan D., Booth I.R. (1993). Activation of potassium channels during metabolite detoxification in *Escherichia coli*. Mol. Microbiol..

[169] MacLean M.J., Ness L.S., Ferguson G.P., Booth I.R. (1998). The role of glyoxalase I in the detoxification of methylglyoxal and in the activation of the KefB K^+^ efflux system in *Escherichia coli*. Mol. Microbiol..

[170] Chakraborty S., Chaudhuri D., Balakrishnan A., Chakrawortty D. (2014). *Salmonella* methylglyoxal detoxification by *STM3117*-encoded lactoylglutathione lyase affects virulence in coordination with *Salmonella* pathogenicity island 2 and phagosomal acidification. Microbiology.

[171] Ozyamak E., Black S.S., Walker C.A., MacLean M.J., Bartlett W., Miller S., Booth I.R. (2010). The critical role of S-D-lactoylglutathione formation during methylglyoxal detoxification in *Escherichia coli*. Mol. Microbiol..

[172] Ness L., Booth I.R. (1999). Different foci for the regulation of the activity of the KefB and KefC glutathione-gated K^+^ efflux systems. J. Biol. Chem..

[173] McKie J.H., Douglas K.T. (1993). Structural relationships between glyoxalase I and membrane transport proteins. Biochem. Soc. Transact..

[174] Funderburk L.H., Aldwin L., Jencks W.P. (1978). Mechanisms of general acid and base catalysis of the reactions of water and alcohols with formaldehyde. J. Am. Chem. Soc..

[175] Morrissey A., Rosner E., Lanning J., Parachuru L., Chowdhury P.D., Han S., Lopez G., Tong X.Y., Yoshida H., Nakamura T.Y. (2015). Immunolocalization of K_ATP_ channel subunits in mouse and rat cardiac myocytes and the coronary vasculature. BMC Physiol..

[176] Nichols C.G. (2006). K_ATP_ channels as molecular sensors of cellular metabolism. Nature.

[177] Kalapos M.P. (2007). Possible mechanism for the effect of ketogenic diet in cases of uncontrolled seizures / Reconsideration of acetone theory. Med. Hypotheses.

[178] Yang Y., Shi W., Cui N., Wu Z., Jiang C. (2010). Oxidative stress inhibits vascular K_ATP_ channels by S-Glutathionylation. J. Biol. Chem..

[179] Yang Y., Shi W., Chen X., Cui N., Konduru A.S., Shi Y., Trower T., Zhang S., Jiang C. (2011). Molecular basis and structural insight of vascular K_ATP_ channel gating by S-Glutathionylation. J. Biol. Chem..

[180] Yang Y., Li S., Konduru A.S., Zhang S., Trower T., Shi W., Cui N., Yu L., Wang Y., Zhu D. (2012). Prolonged exposure to methylglyoxal causes disruption of vascular K_ATP_ channel by mRNA instability. Am. J. Physiol. Cell Physiol..

[181] Yang Y., Konduru A.S., Cui N., Trower T.C., Shi W., Shi Y., Jiang C. (2014). Acute exposure of methylglyoxal leads to activation of K_ATP_ channels expressed in HEK293 cells. Acta Pharmacol. Sin..

[182] Mukohda M., Yamawaki H., Nomura H., Okada M., Hara Y. (2009). Methylglyoxal inhibits smooth muscle contraction in isolated blood vessels. J. Pharmacol. Sci..

[183] Li S.-S., Wu Y., Jin X., Jiang C. (2015). The SUR2B subunit of rat vascular K_ATP_ channel is targeted by miR-9a-39 induced by prolonged exposure to methylglyoxal. Am. J. Physiol. Cell Physiol..

[184] Wang Y., Hall L.M., Kujawa M., Li H., Zhang X., O’Meara M., Ichinose T., Wang J.M. (2019). Methylglyoxal triggers human aortic endothelial cell dysfunction via modulation of the K_ATP_/MAPK pathway. Am. J. Physiol. Cell Physiol..

[185] Szabó I., Leanza L., Gulbins E., Zoratti M. (2012). Physiology of potassium channels in the inner membrane of mitochondria. Pflüg. Arch. Eur. J. Physiol..

[186] Szewczyk A., Jarmuszkiewicz W., Kunz W.S. (2009). Mitochondrial potassium channels. IUBMB Life.

[187] Malinska D., Mirandola S.R., Kunz W.S. (2010). Mitochondrial potassium channels and reactive oxygen species. FEBS Lett..

[188] O’Rourke B., Cortassa S., Aon M.A. (2005). Mitochondrial ion channels: Gatekeepers of life and death. Physiology.

[189] Laskowski M., Augustynek B., Kuliwak B., Koprowski P., Bednarczyk P., Jarmuszkiewicz W., Szewczyk A. (2016). What do we know about mitochondrial potassium channels?. Biochim. Biophys. Acta.

[190] Hooper N.I., Tisdale M.J., Thornalley P.J. (1987). Glyoxalase activity during differentiation of human leakaemia cells in vitro. Leuk. Res..

[191] Clelland J.D., Allen R.E., Thornalley P.J. (1992). Inhibition of growth of human leukaemia 60 cells by S-2-hydroxyacylglutathiones and monoethyl ester derivatives. Biochem. Pharmacol..

[192] Principato G.P., Bodo M., Biagioni M.G., Rosi G., Liotti F.S. (1982). Glyoxalases and glutathione reductase activity changes in chicken liver during embryo development and after hatching. Acta Embryol. Morphol. Exp..

[193] Dixit A., Garg L.C., Sutrave P., Rao A.R. (1983). Glyoxalase I in regenarating mouse liver exposed to carcinogens. Biochem. Intern..

[194] Principato G.B., Locci P., Rosi G., Talesa V., Giovannini E. (1983). Activity changes of glyoxalases I-II and glutathione reductase in regenerating rat liver. Biochem. Intern..

[195] Dudani A.K., Srivastava L.K., Prasad R. (1984). Glyoxalase-I activity and cell cycle regulation in yeast. Biochem. Biophys. Res. Commun..

[196] Bruschelli G., Mariucci G., Principato G.B., Lioti F.S. (1986). Glyoxalase activity in quiescent and proliferating human fibroblasts. Cell Mol. Biol..

[197] Matsuura T., Owada K., Sano M., Saito S., Tomita I., Ikekawa T. (1986). Studies on methylglyoxal II/Changes of methylglyoxal level accompanying the changes of glyoxalase I and II activities in mice bearing L1210 leukemia and sarcoma 180. Chem. Pharmacol. Bull..

[198] Basu A., Sethi U., Guha-Mukherjee S. (1988). Induction of cell division in leaf cells of Coconut Palm by alteration of pH and its correlation with glyoxalase-I activity. J. Exp. Bot..

[199] Chakravarty T.N., Sopory S.K. (1998). Blue light stimulation of cell proliferation and glyoxalase I activity in callus cultures of Amaranthus paniculatus. Plant Sci..

[200] Kalia S., Pal S., Guha-Mukherjee S. (1998). Activation of glyoxalase I during the cell division cycle and its homology with auxin regulated genes. Plant Sci..

[201] Gillespie E. (1978). Concanavalin A increases glyoxalase enzyme activities in polymorphonuclear leukocytes and lymphocytes. J. Immunol..

[202] Gillespie E. (1979). Effects of S-D-lactoylglutathione and inhibitors of glyoxalase I on histamine release from human leukocytes. Nature.

[203] Oliver J.M., Albertini D.F., Berlin R.D. (1976). Effects of glutathione-oxidizing agents on microtubule assembly and microtubule-dependent surface properties of human neutrophils. J. Cell Biol..

[204] Etienne-Manneville S. (2004). Actin and microtubules in cell motility: Which one is in control?. Traffic.

[205] Riesco A., Santos-Buitrago B., De Las Rives J., Knapp M., Santos-Garcia G., Talcott C. (2017). Epidermal growth factor signaling towards proliferation: Modelling and logic interference using forward and backward search. BioMed Res. Int..

[206] Rani R., Kumar S., Sharma A., Mohanty S.K., Donnelly B., Tiao G.M., Gandhi C.R. (2018). Mechanisms of concanavalin A-induced cytokine synthesis by hepatic stellate cells: Distinct roles of interferon regulatory factor-1 in liver injury. J. Biol. Chem..

[207] Thornalley P.J., Greskowiak M., Della Bianca V. (1989). Potentiation of secretion from neutrophils by S-D-lactoylglutathione. Med. Sci. Res..

[208] Allen R., Thornalley P.J. (1993). The effect of S-D-lactoylglutathione on the movement of neutrophils. Biochem. Soc. Transact..

[209] Kalapos M.P. (2011). S-D-lactoylglutathione as a potential state marker for hemolysis. Med. Hypotheses.

[210] Wang D., Li W., Yin L., Du Y., Zhang S., Suo J. (2020). Association of serum levels of deoxyribose 1-phosphate and S-lactoylglutathione with neoadjuvant chemotherapy sensitivity in patients with gastric cancer: A metabolomics study. Oncol. Lett..

[211] Doğan H.F., Şenol O., Bolat S., Yıldız S.N., Büyüktuna S.A., Sarıismailoğlu R., Doğan K., Hasbek M., Hekim S.N. (2021). Understanding the pathophysiological changes via untargeted metabolomics in COVID-19 patients. J. Med. Virol..

[212] Kalapos M.P. (2008). Methylglyoxal and glucose metabolism: A historical perspective and future avenues for research. Drug Metab. Drug Interact..

[213] Kalapos M.P. (2007). Can ageing be prevented by dietary restriction?. Mech. Aging Dev..

[214] Braun L., Garzó T., Riba P., Mandl J., Kalapos M.P. (1994). Methylglyoxal and cell viability. Int. J. Biochem..

[215] Karg E., Németh I., Horányi M., Pintér S., Vécsei L., Hollán S. (2000). Diminished blood levels of reduced glutathione and α-tocopherol in two triose-phosphate isomerase-deficient brothers. Blood Cells Mol. Dis..

[216] Van Wijk R., van Solinge W.W. (2005). The energy-less red blood cells is lost: Erythrocyte enzyme abnormalities of glycolysis. Blood.

[217] Orosz F., Oláh J., Ovádi J. (2006). Triose-phosphate isomerase deficiency: Facts and doubts. IUBMB Life.

[218] Ahmed N., Battah S., Karachalias N., Babaei-Jadidi R., Horányi M., Baróti K., Hollán S., Thornalley P.J. (2003). Increased formation of methylglyoxal and protein glycation, oxidation and nitrosation in triosephosphate isomerase deficiency. Biochim. Biophys. Acta.

[219] Beisswenger P.J., Howell S.K., Smith K., Szwergold B.S. (2003). Glyceraldehyde-3-phosphate dehydrogenase activity is an independent modifier of methylglyoxal levels in diabetes. Biochim. Biophys. Acta.

[220] Beisswenger P.J., Howell S.K., Nelson R.G., Mauer M., Szwergold B.S. (2003). α-Oxoaldehyde metabolism and diabetic complications. Biochem. Soc. Transact..

[221] Beisswenger P.J., Drummond K.S., Nelson R.G., Howell S.K., Szwergold B.S., Mauer M. (2005). Susceptibility to diabetic nephropathy is related to dicarbonyl and oxidative stress. Diabetes.

[222] Valentine W.N. (1975). Metabolism of human erythrocytes. Arch. Intern. Med..

[223] Agar N.S., Board P.G., Bell K. (1984). Studies of erythrocyte glyoxalase II in various domestic species: Discovery of glyoxalase II deficiency in the horse. Anim. Blood Groups Biochem. Genet..

[224] Valentine W.N., Paglia D.E., Neerhout R.C., Konrad P.N. (1970). Erythrocyte glyoxalase II with coincidental hereditary elliptocytosis. Blood.

[225] Jerzykowski T., Winter R., Matuszewski W., Piskorska D. (1978). The re-evaluation of studies on the distribution of glyoxalases in animal and tumour tissues. Int. J. Biochem..

[226] Opperdoes F.R., Michels P.A.M., Myler P.J., Fasel V. (2003). The metabolic repertoire of Leshmania and implications for drug discovery. Leishmania: After the Genome.

[227] Talesa V.N., Ferri I., Bellezza G., Love H.D., Sidoni A., Antognelli C. (2017). Glyoxalase 2 is involved in human prostate cancer progression as part of a mechanism driven by PTEN/P13K/AKT/mTOR signaling with involvement of PKM2 and Erα. Prostate.

[228] Ward G.M., Harrison L.C. (1986). Structure of the human erythrocyte insulin receptor. Diabetes.

[229] Joost H.-G., Bell G.I., Best J.D., Birnbaum M.J., Charron M.J., Chen Y.T., Doege H., James D.E., Lodish H.F., Moley K.H. (2002). Nomenclature of the GLUT/SLC2A family of sugar/polyol transport facilitators. Am. J. Physiol. Endocrinol. Metab..

[230] Thornalley P.J., Jahan I., Ng R. (2001). Suppression of the accumulation of triose-phosphates and increased formation of methylglyoxal in human red blood cells during hyperglycemia by thiamine in vitro. J. Biochem..

[231] Lee D.-Y., Lin Y.-C., Chang G.-D. (2021). Biochemical regulation of the glyoxalase system in response to insulin signaling. Antioxidants.

[232] Kalapos M.P., Riba P., Garzó T., Mandl J. (1996). Glucose formation from methylglyoxal in hepatocytes from streptozotocin-induced diabetic mice: The effect of insulin. Experientia.

[233] Kalapos M.P. (2013). Where does plasma methylglyoxal originate from?. Diabetes Res. Clin. Pract..

[234] Atkins T.W., Thornalley P.J. (1989). Erythrocyte glyoxalase activity in genetically obese (ob/ob) and streptozotocin diabetic mice. Diabetes Res..

[235] Vogt-Moller P. (1929). Ist Avitaminosis B1 eine Intoxikation mit Methylglyoxal?. Biochem. Z.

[236] Alonso J., Angermeyer M.C., Bernert S., Bruffaerts R., Brugha T.S., Bryson H., de Girolamo G., Graaf R., Demyttenaere K., Gasquet I. (2004). 12-Month comorbidity patters and associated factors in Europe: Results from the European Study of the Epidemiology of Mental Disorders (ESMeD) project. Acta Psychiatr. Scand..

[237] Lieber C.S. (2000). Hepatic, metabolic, and nutritional disorders of alcoholism: From pathogenesis to therapy. Crit. Rev. Clin. Lab. Sci..

[238] Kalapos M.P. (2007). Introduction to Alcohology.

[239] Lonsdale D. (2006). A review of the biochemistry, metabolism and clinical benefits of thiamin(e) and its derivatives. eCAM.

[240] Kalapos M.P. (2003). On the mammalian acetone metabolism/From chemistry to clinical implications. Biochim. Biophys. Acta.

[241] Beisswenger B.G.K., Delucia E.M., Lapoint N., Sanford R.J., Beisswenger P.J. (2005). Ketosis leads to increased methylglyoxal production on the Atkins diet. Ann. N. Y. Acad. Sci..

[242] Weber Y.G., Lerche H. (2009). Genetics of Paroxysmal Dyskinesias. Curr. Neurol. Neurosci. Rep..

[243] Shen Y., Ge W.-P., Li Y., Hirano A., Rohlmann A., Missler M., Tsien R.W., Jan L.Y., Fu Y.-H., Ptáĉek L.J. (2015). Protein mutated in paroxysmal dyskinesia interacts with the active zone protein RIM and suppresses synaptic vesicle exocytosis. Proc. Natl. Acad. Sci. USA.

[244] Vermeulen N., Vermiere S., Arijs I., Michiels G., Ballet V., Derua R., Waelkens E., Van Lommel L., Schuit F., Rutgeerts P. (2011). Seroreactivity against glycolytic enzymes in inflammatory Bowel Disease. Inflamm. Bowel Dis..

[245] Ritter K., Brestrich H., Nellen B., Kratzin H., Eiffer H., Thomssen R. (1990). Autoantibodies against triose-phosphate isomerase/A Possible Clue to Pathogenesis of Hemolytic Anemia in Infectious Mononucleosis. J. Exp. Med..

[246] Kültz D. (2005). Molecular and evolutionary basis of the cellular stress response. Annu. Rev. Physiol..

[247] Yadav S.K., Singla-Pareek S.L., Ray M., Reddy M.K., Sopory S.K. (2005). Methylglyoxal levels in plants under salinity stress are dependent on glyoxalase I and glutathione. Biochem. Biophys. Res. Commun..

[248] Russell J.B. (1993). Glucose toxicity in *Prevotella ruminicola*: Methylglyoxal accumulation and its effect on membrane physiology. Appl. Environ. Microbiol..

[249] Kalapos M.P. (2008). The tandem of free radicals and methylglyoxal. Chem.-Biol. Interact..

[250] Abordo E.A., Minhas H.S., Thornalley P.J. (1999). Accumulation of α-oxoaldehydes during oxidative stress: A role in cytotoxicity. Biochem. Pharmacol..

[251] Eberhardt M.J., Filipovic M.R., Leffler A., de la Roche J., Kistner K., Fisher M.J., Fleming T., Zimmerma K., Ivanovic-Burmazovic I., Nawroth P.P. (2012). Methylglyoxal activates nociceptors through transient receptor potential channel A1 (TRPA1)/A possible mechanism of metabolic neuropathies. J. Biol. Chem..

[252] Kwak M.-K., Ku M.H., Kang S.-O. (2014). NAD^+^-linked alcohol dehydrogenase 1 regulates methylglyoxal concentration in *Candida albicans*. FEBS Lett..

[253] Kaur C., Sharma S., Singla-Pareek S.L., Sopory S.K. (2016). Methylglyoxal detoxification in plants: Role of glyoxalase pathway. Indian J. Plant Physiol..

[254] Fierro C., López-Cristolfanini C., Latorre N., Rivas J., Contreras-Porcia L. (2016). Methylglyoxal metabolism in seaweeds during desiccation. Rev. Biol. Mar. Oceanogr..

[255] Stratmann B., Goldstein B., Thornalley P.J., Rabbani N., Tschoepe D. (2017). Intracellular accumulation of methylglyoxal by glyoxalase 1 knock down alters collagen homeostasis in L6 myoblasts. Int. J. Mol. Sci..

[256] Inoue Y., Kimura A. (1999). Glycolytic-methylglyoxal pathway Molecular evolution and stress response of glyoxalase I in Saccharomyces cerevisiae. Proc. Jpn. Acad. Ser. B Phys. Biol. Sci..

[257] Espartero J., Sanchez-Aquayo I., Pardo J.M. (1995). Molecular characterization of glyoxalase I from a higher plant: Upregulation by stress. Plant Mol. Biol..

[258] Inoue Y., Tsujimoto Y., Kimura A. (1998). Expression of the glyoxalase I gene of *Saccharomyces cerevisiae* is regulated by high osmolarity glycerol mitogen-activated protein kinase pathway in osmotic stress response. J. Biol. Chem..

[259] Choudhary D., Chandra D., Lochab S.P., Sarma A., Kale R.K. (1999). Response of the glyoxalase system to low doses of mixed radiation. Phys. Med..

[260] Yadav S.K., Singla-Pareek S.L., Reddy M.K., Sopory S.K. (2005). Transgenic tobacco plants overexpressing glyoxalase enzymes resist an increase methylglyoxal and maintain higher reduced glutathione levels under salinity stress. FEBS Lett..

[261] Takatsume Y., Izawa S., Inoue Y. (2003). Unique regulation of glyoxalase I activity during osmotic stress response in fission yeast *Schizosaccharomíces pombe*: Neither the mRNA nor the protein level of glyoxalase I increase under conditions that enhance its activity. Arch. Microbiol..

[262] Hossain M.A., Hasanuzzaman M., Fujita M. (2010). Up-regulation of antioxidant and glyoxalase systems by exogenous glycinebetaine and proline in mung bean confer tolerance to cadmium. Physiol. Mol. Biol. Plants.

[263] (1988). Pharmaceuticals Containing S-lactoyl-glutathione and/or Its Salt as Active Ingredient. Japanese Patent.

[264] Schumacher D., Morgenstern J., Oguchi Y., Volk N., Kopf S., Groener J.B., Nawroth P.P., Fleming T., Freichel M. (2018). Compensatory mechanisms for methylglyoxal detoxification in experimental and clinical diabetes. Mol. Metab..

[265] Morcos M., Du X.-H., Pfistere F., Hutter H., Sayed A.A.R., Thornalley P., Ahmed N., Baynes J., Thorpe S., Kukudov G. (2008). Glyoxalae-1 prevents mitochondrial protein modification and enhances lifespan in *Caenorhabditis elegans*. Aging Cell.

[266] Schlotterer A., Kukudov G., Bozorgmehr F., Hutter H., Du X.-H., Oikonomou D., Ibrahim Y., Pfisterer F., Rabbani N., Thornalley P. (2009). *C. elegans* as model for the study of high glucose-mediated life span reduction. Diabetes.

[267] Scheckhuber C.Q., Mack S.J., Strobel I., Ricciardi F., Gispert S., Osiewacz H.D. (2010). Modulation of the glyoxalase system in the aging model Podospora anserina: Effects on growth and lifespan. Aging.

[268] Giacco F., Du X.-H., D’Agatti V.D., Milne R., Sui G.-S., Geoffrion M., Brownlee M. (2014). Knockdown of glyoxalase 1 mimics diabetic nephropathy in nondiabetic mice. Diabetes.

[269] Lodd E., Wiggenhauser L.M., Morgenstern J., Fleming T.H., Poschet G., Büttner M., Tabler C.T., Wohlfart D.P., Nawroth P.P., Kroll J. (2019). The combination of loss of glyoxalase1 and obesity results in hyperglycemia. JCI Insight.

[270] Morgenstern J., Fleming T., Schumacher D., Eckstein V., Freichel M., Herzig S., Nawroth P. (2017). Loss of glyoxalase 1 induces compensatory mechanism to achieve dicarbonyl detoxification in mammalian Schwann cells. J. Biol. Chem..

[271] Schwalwijk C.G., Steheower C.D.A. (2020). Methylglyoxal, a highly reactive dicarbonyl compound, in diabetes, its vascular complications, and other age related diseases. Physiol. Rev..

[272] Ayoub F., Zaman M., Thornalley P.J., Masters J. (1993). Glyoxalase activities in human tumour cell lines in vitro. Anticancer Res..

